# Overdose Prevention Centers, Crime, and Disorder in New York City

**DOI:** 10.1001/jamanetworkopen.2023.42228

**Published:** 2023-11-13

**Authors:** Aaron Chalfin, Brandon del Pozo, David Mitre-Becerril

**Affiliations:** 1 National Bureau of Economic Research, University of Pennsylvania, Philadelphia; 2 Division of General Internal Medicine, Rhode Island Hospital, The Warren Alpert Medical School of Brown University, Providence; 3 University of Connecticut School of Public Policy, Hartford

## Abstract

**Importance:**

The first government-sanctioned overdose prevention centers (OPCs) in the US opened in New York City (NYC) in November 2021 amid concerns that they may increase crime and disorder, representing a significant political challenge to OPCs.

**Objective:**

To identify whether opening the first 2 government-sanctioned OPCs in the US was associated with changes in crime and disorder.

**Design, Setting, and Participants:**

In this cohort study, difference-in-differences Poisson regression models were used to compare crime, residents’ requests for assistance for emergencies and nuisance complaints, and police enforcement in the vicinity of NYC’s 2 OPCs with those around 17 other syringe service programs that did not offer overdose prevention services from January 1, 2019, through December 31, 2022.

**Main Outcomes and Measures:**

Changes in the volume of crimes reported by the public or observed by police; arrests for drug possession and weapons; 911 calls and 311 calls regarding crime, public nuisances, and medical events; and summonses issued by police for criminal infractions in both the immediate vicinity of the sites (ie, a hexagonal area spanning about 6 city blocks) and their wider neighborhoods (ie, a tesselated 3-hexagon array spanning about 18 city blocks).

**Results:**

No significant changes were detected in violent crimes or property crimes recorded by police, 911 calls for crime or medical incidents, or 311 calls regarding drug use or unsanitary conditions observed in the vicinity of the OPCs. There was a significant decline in low-level drug enforcement, as reflected by a reduction in arrests for drug possession near the OPCs of 82.7% (95% CI, −89.9% to −70.4%) and a reduction in their broader neighborhoods of 74.5% (95% CI, −87.0% to −50.0%). Significant declines in criminal court summonses issued in the immediate vicinity by 87.9% (95% CI, −91.9% to −81.9%) and in the neighborhoods around the OPCs by 59.7% (95% CI, −73.8% to −38.0%) were observed. Reductions in enforcement were consistent with the city government’s support for the 2 OPCs, which may have resulted in a desire not to deter clients from using the sites by fear of arrest for drug possession.

**Conclusions and Relevance:**

In this difference-in-differences cohort study, the first 2 government-sanctioned OPCs in the US were not associated with significant changes in measures of crime or disorder. These observations suggest the expansion of OPCs can be managed without negative crime or disorder outcomes.

## Key Points

**Question:**

What trends in crime and disorder were associated with the opening of 2 overdose prevention centers (OPCs) in New York City (NYC) in November 2021?

**Findings:**

This cohort study of 2 OPCs and 17 syringe service programs found no significant increases in crimes recorded by the police or calls for emergency service in NYC neighborhoods where 2 OPCs were located. Consistent with the city’s commitment to ensuring clients could use the centers free from law enforcement interference, large, statistically significant declines in police narcotics enforcement around the OPCs were observed.

**Meaning:**

These findings suggest that concerns about crime and disorder remain substantial barriers to the expansion of OPCs in US cities, and initial data from NYC do not support these concerns.

## Teaser

This cohort study uses administrative data to evaluate the changes in crime and disorder associated with the opening of 2 overdose prevention centers in New York City.

## Introduction

Overdose prevention centers (OPCs), also termed *safe injection* or *safe consumption sites*, are facilities where individuals consume illicit drugs under the observation of trained staff to mitigate risk of fatal overdose. The first officially acknowledged OPC was opened in the Netherlands in the 1970s, and OPCs have since proliferated throughout Europe, Canada, and Australia.^1,2^ By intervening with use of naloxone, oxygen, and other overdose mitigation techniques, OPCs prevent fatal opioid overdoses on their premises.^3,4^

Amid an unprecedented overdose crisis,^5^ the US has been hesitant to implement OPCs as part of its fatal overdose prevention strategy; until recently, the handful of domestic OPCs operated clandestinely.^6,7^ Concerns persist that they openly sanction criminalized activity^8,9^ and that people who use drugs at OPCs might generate additional crime and nuisance conditions. Given that locations selected for OPCs often experience sociodemographic conditions that leave them more vulnerable to these problems,^10,11^ these concerns have resulted in substantial political opposition,^12,13^ similar to syringe service programs (SSPs) when they were introduced in the 1980s.^14,15^

In late November 2021, public officials in New York City (NYC) announced that 2 OPCs had commenced operations, making them the first officially sanctioned sites in the US.^16^ By February 2023, more than 2300 clients had visited the centers approximately 55 000 times, requiring more than 700 overdose interventions with no fatalities.^17^ While these data suggest OPCs are well equipped to reduce the risk of fatal overdoses, their effects on crime and disorderly behavior have yet to be evaluated.

As plans to open OPCs proceed in Rhode Island, Massachusetts, and elsewhere,^17^ lawmakers in Pennsylvania passed a preemptive measure effectively banning them^18,19^ due to concerns that include increased crime and disorder.^20^ In NYC, the federal prosecutor for Manhattan has characterized the city’s OPCs as unlawful and threatened to close them.^21^ Officials tasked with implementing effective public health strategies for reducing fatal overdoses would therefore benefit from an evaluation of the changes in crime and disorder associated with opening OPCs in a US setting.^22^ This study used administrative data to conduct such an evaluation.

## Methods

### Study Setting

This difference-in-differences cohort study evaluated the first 2 government-sanctioned OPCs in the US, which opened in NYC on November 30, 2021. Operated by OnPoint NYC, a harm reduction coalition, they operate in the Manhattan communities of East Harlem and Washington Heights, at the locations of long-established SSPs that provide access to naloxone, harm reduction resources, and linkages to treatment for substance use disorder and infectious diseases. Our primary comparison group consisted of the 17 other state-authorized brick-and-mortar SSPs located throughout NYC that operated at least 3 days a week and that have not, to date, offered on-site overdose prevention services.^23^ We also constructed 2 alternative comparison groups of locations with similar levels of serious felony crime reports and drug arrests during the 2 years before the OPCs began operating. We followed the combined checklist in the Strengthening the Reporting of Observational Studies in Epidemiology (STROBE) reporting guideline as appropriate for a difference-in-differences study. This study did not constitute human subjects research and is exempt from institutional review board review and informed consent requirements per the Common Rule.^24,25^

### Data Sources

We leveraged 5 administrative data sets that were placed in the public domain by the City of New York and are available via the NYC Open Data portal.^26^ These included criminal complaints, arrest reports, criminal court summonses, 911 call records, and 311 call records from January 1, 2019, through December 31, 2022. Criminal complaints reflect public reports of crimes to law enforcement and incidents independently observed by police. Arrest reports record both proactive arrests made by the NYC Police Department and those made after a person has allegedly committed a crime. Both arrest and complaint reports contain the type of crime alleged and its date, time, and location of occurrence. Criminal court summonses are tickets issued by police for minor infractions that include crime and violations such as drug possession, trespassing, and public alcohol consumption. The city’s 911 and 311 records document calls regarding criminal incidents, medical events, and quality of life concerns such as discarded syringes, illicit drug use, trespass, homelessness, unsanitary conditions, excessive noise, and other reports of disorder. The city’s 911 system is used to report emergencies, and the 311 system is used to report nonemergency concerns. All records provide latitude and longitude coordinates to the nearest midblock or intersection of the incident.

### Approach

**Figure 1.**
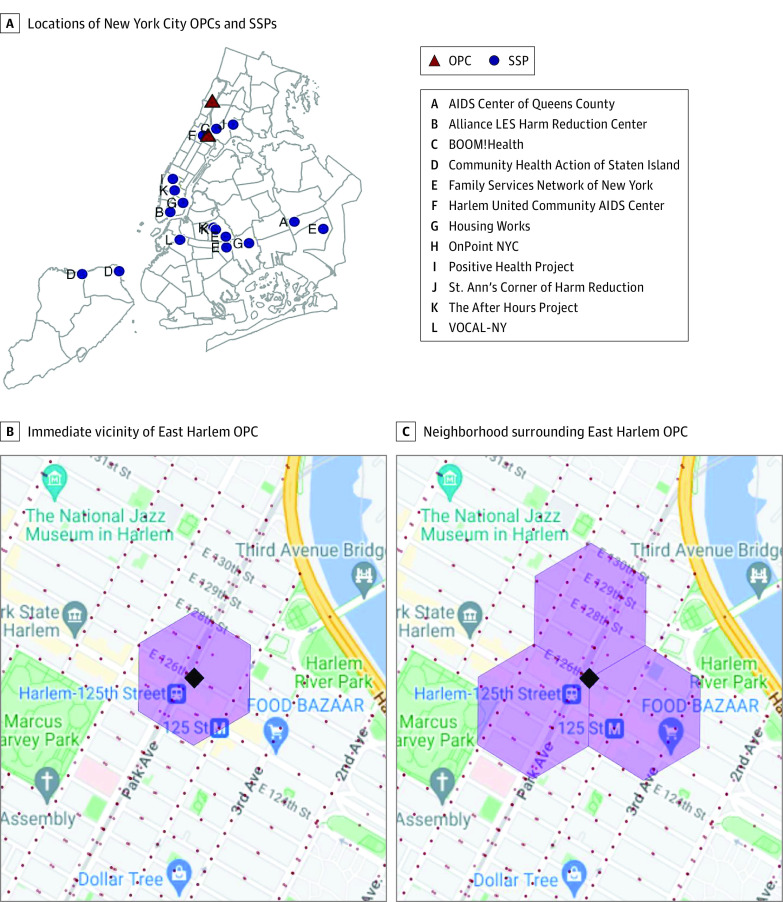
Locations of Overdose Prevention Centers (OPCs) and Syringe Service Programs (SSPs) in New York City A, New York City map excerpt indicating the locations of the 2 OPCs and the 17 SSPs. The solid gray lines demarcate police precinct boundaries. B, Hexagon indicating the immediate vicinity of the OPC in East Harlem. C, Tessellated hexagonal array indicating the neighborhood surrounding the East Harlem OPC.

We identified changes in crime and disorder associated with the opening of OPCs using a difference-in-differences regression framework in which we compared changes in outcomes around the 2 OPCs with the changes around comparison sites. In our primary models, comparison sites consisted of the 17 SSPs that did not provide on-site overdose prevention. A map of the 2 OPCs and the 17 comparison sites is provided in Figure 1. Our analysis considered 2 aggregate crime categories (violent and property crimes), 2 categories of proactive arrests (drug and weapons possession), 8 types of public calls for service (911 calls regarding criminal activity, trespass, and medical emergencies and 311 calls regarding complaints about drug use, unsanitary conditions, excessive noise, abandoned vehicles, and homelessness), and police issuance of criminal court summonses.

New York City’s 2 OPCs opened on November 30, 2021. Our preintervention period was January 1, 2019, to November 29, 2021, and our postintervention period was December 1, 2021, through December 31, 2022. Spatial analyses of crime and disorder were conducted in the immediate vicinity of OPCs and comparison sites and, to assess possible spillover effects, in the wider neighborhoods surrounding these sites. Following the methods of prior studies with a similar objective,^27,28,29^ we divided the city into discrete areas by overlaying a block-level map of NYC with a tessellated hexagonal array, centering the hexagons to demarcate areas around OPCs and comparison sites. Hexagons were used because they can be tessellated without geographic omission or the need for alteration and have the least internal variation in their radii. For analyses of the immediate vicinity, each site was placed at the geographic center of a hexagon spanning about 6 city blocks (22 acres; 88 919 m^2^); for analyses of broader neighborhoods, sites were placed at the center of a tesselated 3-hexagon array spanning about 18 city blocks (66 acres; 266 757 m^2^). Figure 1B and C offer an example using the East Harlem OPC. Aggregate population-weighted sociodemographic zip code–level data for the neighborhoods around the OPCs and comparison SSPs are presented in eTable 8 in Supplement 1.

### Statistical Analysis

Data were aggregated to the hexagon monthly level, and Poisson regression models were used to estimate relative changes in crime and disorder between the 2 OPCs and comparison sites. The models, which are detailed in the eMethods in Supplement 1, condition on site and on month-year fixed effects that account for time-invariant differences between areas and citywide time trends. Significance was prespecified using 2-tailed 95% CIs. To account for arbitrary serial correlation and heteroskedasticity in the regression errors, SEs were clustered at the hexagon level. As a quasi-experimental approach, difference-in-differences models permit causal inference under an assumption of parallel preintervention and counterfactual trends across areas with OPCs and comparison sites. Using a similar count data regression described in the eMethods in Supplement 1, we empirically verified that intervention and comparison sites were experiencing common trends in key outcomes prior to the OPCs commencing operation.

For each outcome variable, we calculated the raw Poisson coefficient and SE and its percentage change. To provide context for percentage changes, we also calculated the preintervention mean of the 2 OPCs. Sensitivity analyses are presented in the eMethods in Supplement 1. They include estimates arising from an alternative comparison group, estimates that additionally condition on preintervention trends and police patrol borough linear time trends, and estimates from negative binomial regressions. We used R statistical software, version 4.2.0 (R Project for Statistical Computing) for all analyses. The data we used are in the public domain and did not require adjustments or deletions for missing values.^26^

## Results

### Descriptive Statistics

From January 1, 2019, through December 31, 2022, across the 2 OPCs and the 17 comparison sites, there were 4414 serious criminal complaints (monthly hexagon mean [SD], 4.8 [5.0]), 138 402 calls to 911 (monthly hexagon mean [SD], 151.7 [135.3]), 20 876 calls to 311 (monthly hexagon mean [SD], 22.9 [24.8]), and 2123 criminal court summonses (monthly hexagon mean [SD], 2.3 [4.4]). The mean number of monthly incidents by subtype in the immediate vicinity of the sites before and after the intervention are presented in Table 1. Overall, felonies rose at both OPC locations by 21.0% (mean [SD], 6.7 [4.5] to 8.1 [5.5]) and at SSP comparison locations by 18.0% (mean [SD], 4.4 [4.7] to 5.2 [5.5]), which is consistent with the general rise in crime in NYC during the study period, an increase predominantly driven by property crime.^30^

**Table 1.** Descriptive Statistics and Hexagon Monthly Level Data by Intervention Period^a^

	Neighborhoods with OPCs (n = 2)		Neighborhoods with SSPs (n = 17)	
	Preintervention	Postintervention	Preintervention	Postintervention
Crimes				
Index^b^	6.7 (4.5)	8.1 (5.5)	4.4 (4.7)	5.2 (5.5)
Violent	7.7 (5.6)	7.5 (5.2)	3.9 (4.1)	4.1 (4.4)
Murder	0.1 (0.3)	0.1 (0.3)	0.02 (0.2)	0.1 (0.8)
Robbery	1.7 (1.8)	1.6 (1.6)	0.7 (1.1)	0.9 (1.3)
Aggravated assault	1.9 (2.1)	2.7 (2.6)	0.9 (1.4)	1.0 (1.4)
Simple assault	4.1 (3.4)	3.1 (2.0)	2.2 (2.5)	2.1 (2.3)
Property	3.1 (2.3)	3.7 (2.6)	2.7 (3.3)	3.2 (3.8)
Burglary	0.6 (1.0)	0.6 (0.9)	0.6 (1.1)	0.6 (1.1)
Theft	2.3 (2.0)	2.6 (2.2)	1.9 (2.7)	2.3 (2.9)
Motor vehicle theft	0.2 (0.5)	0.5 (0.8)	0.2 (0.5)	0.3 (0.6)
Law enforcement				
Weapons arrests^c^	1.0 (1.5)	0.6 (0.8)	0.3 (0.8)	0.4 (0.9)
Drug arrests^d^	17.9 (22.6)	5.5 (7.4)	1.2 (2.7)	2.2 (5.7)
Criminal summons	4.9 (8.0)	0.8 (1.4)	2.0 (3.4)	2.6 (5.0)
911 Calls				
Crime^e^	152.0 (85.2)	105.2 (16.4)	106.8 (94.5)	103.4 (92.0)
Assault^f^	10.3 (7.0)	6.0 (2.7)	5.9 (5.6)	5.2 (4.4)
Trespass^f^	2.2 (1.8)	1.6 (1.3)	1.6 (2.5)	1.6 (2.0)
Medical^g^	90.4 (66.0)	44.6 (12.3)	39.1 (43.1)	35.7 (46.9)
311 Calls				
Drug-related^h^	0.5 (0.9)	1.3 (3.1)	0.3 (1.0)	0.4 (0.9)
Unsanitary conditions^i^	3.1 (3.3)	2.6 (2.9)	3.7 (4.2)	3.0 (4.3)
Abandoned vehicle^j^	0.1 (0.4)	0.8 (1.5)	0.4 (1.4)	0.5 (0.8)
Noise complaint^j^	16.1 (17.0)	20.7 (9.6)	15.6 (19.3)	17.0 (29.7)
Homelessness-related^k^	4.1 (5.3)	2.1 (2.8)	2.1 (5.2)	3.4 (8.3)

Abbreviations: OPC, overdose prevention center; SSP, syringe services program.

^a^ Data are from the hexagon-shaped area encompassing the OPCs and SSPs and are presented as the mean (SD) No. of monthly incidents. The preintervention period was January 1, 2019, to November 29, 2021; the postintervention period was December 1, 2021, through December 31, 2022.

^b^ Includes the 6 Uniform Crime Reporting Program part I crimes (murder, robbery, aggravated assault, burglary, theft, and motor vehicle theft).

^c^ Refers to criminal possession of a weapon.

^d^ Refers to the unlawful sale or possession of drugs.

^e^ Includes calls in which a possible crime was in progress or one had been committed.

^f^ These offenses are explicitly mentioned in the call.

^g^ Includes calls requiring the response of an ambulance or fire department medical personnel.

^h^ Includes drug and alcohol activity and discarded syringe calls.

^i^ Includes calls related to rodents, graffiti, dirty and unsanitary conditions, and urinating in public.

^j^ Constitutes quality-of-life calls handled by the New York City Police Department.

^k^ Includes calls related to assisting a person experiencing homelessness, encampments, and homelessness-related street conditions.

Around the 2 OPCs, monthly mean (SD) 911 calls for crime and other emergencies decreased 30.1% from 152.0 (85.2) to 105.2 (16.4) while remaining approximately flat in the comparison locations (106.8 [94.5] vs 103.4 [92.0]; decrease of 3.1%); 911 calls for medical emergencies decreased around the OPCs by 50.1% (mean [SD], 90.4 [66.0] to 44.6 [12.3]) while declining by 8.6% around the comparison locations (mean [SD], 39.1 [43.1] to 35.7 [46.9]). Monthly 311 calls for drug activity rose 106.0% around the OPCs (mean [SD], 0.5 [0.9] to 1.3 [3.1]) and 33.3% around the SSP comparison sites (mean [SD], 0.3 [1.0] to 0.4 [0.9]). eFigure 5 in Supplement 1 shows the raw monthly incident counts per site.

### Regression Estimates

#### Recorded Crime

Point estimates indicate complaint reports for violent crime declined by 7.8% (95% CI, −25.8% to 14.5%) and property crime increased by 2.1% (95% CI, −17.0% to 25.6%) in the vicinity of the 2 OPCs (Table 2); more detailed estimates for the individual crime types are available in eTable 1 in Supplement 1. Standard errors become larger for these estimates, precluding confident conclusions about changes in individual crime types. There is evidence of a comparative 30.4% increase in aggravated assaults, offset by a 19.7% decrease in simple assaults. While interpreting these estimates is speculative, we note the distinction between aggravated and simple assaults can be arbitrary, depending on how an incident is reported and the use of police discretion in how it is classified. Neither result was statistically significant, and in each case the SE was approximately 11%. We did not observe significant changes in complaints of violent or property crime in the broader neighborhood, with results similar to the immediate vicinity (Table 3).

**Table 2.** Associations Between Opening OPCs and Public Safety and Disorder in Their Immediate Vicinity^a^

	Crime		Law enforcement			Calls for service		
	Violent^b^	Property^c^	Weapons arrests^d^	Drug arrests^e^	Criminal summons	Crime 911 calls^f^	Medical 911 calls^g^	Nuisance calls^h^
Treatment × postintervention coefficient (SE)^i^	−0.08 (0.11)	0.02 (0.11)	−0.83 (0.38)^j^	−1.76 (0.27)^k^	−2.11 (0.20)^k^	−0.34 (0.33)	−0.61 (0.40)	0.02 (0.20)
Change (95% CI), %^l^	−7.8 (−25.8 to 14.5)	2.1 (−17.0 to 25.6)	−56.5 (−79.4 to −8.1)	−82.7 (−89.9 to −70.4)	−87.9 (−91.9 to −81.9)	−28.5 (−62.3 to 35.6)	−45.9 (−75.2 to 18.1)	2.5 (−30.1 to 50.3)
Preintervention crime count, mean (SD)	7.7 (5.6)	3.1 (2.3)	1.0 (1.5)	17.9 (22.6)	4.9 (8.0)	152.0 (85.2)	90.4 (66.0)	26.0 (16.8)
No. of observations	912	912	912	912	912	912	912	912

Abbreviation: OPC, overdose prevention center.

^a^ Data are from an analysis that encompassed a single hexagon-shaped area around the OPC site.

^b^ Includes murder, robbery, and aggravated and simple assault.

^c^ Includes burglary, theft, and motor vehicle theft.

^d^ Refers to criminal possession of a weapon.

^e^ Refers to the unlawful sale or possession of drugs.

^f^ Refers to calls made to police where there was a possible crime in progress or where one had been committed.

^g^ Includes calls requiring an ambulance or the response of fire department medical personnel.

^h^ Includes 911 calls for trespass and 311 calls about homelessness (assisting a person experiencing homelessness, encampments, and homelessness-related street condition) and disorder (rodents, graffiti, dirty and unsanitary conditions, drug and alcohol activity, public urination, and 311 calls under the New York City Police Department’s jurisdiction [eg, abandoned vehicles and noise complaints]).

^i^ Calculated as difference-in-differences Poisson regression estimates on the association of public safety and the opening of the OPCs. The specifications include hexagon and month-year fixed effects. Robust SEs clustered at the hexagon level in parentheses. The specification follows the equation in the eMethods in Supplement 1, where postintervention is an indicator for whether a given observation occurred after November 30, 2021, when the 2 OPCs were opened to the public. Treatment is an indicator for whether a hexagon contains 1 of the 2 OPCs as opposed to a comparison unit. Hence, Table 2 shows the coefficient on the interaction between postintervention and treat, which is the estimated difference-in-differences intervention effect.

^j^ *P* < .05.

^k^ *P* < .001.

^l^ Calculated as incidence rate ratio − 1 = exp(β) − 1.

**Table 3.** Associations Between Opening OPCs and Public Safety and Disorder in Their Wider Neighborhoods^a^

	Crime		Law enforcement			Calls for service		
	Violent^b^	Property^c^	Weapons arrests^d^	Drug arrests^e^	Criminal summons	Crime 911 calls^f^	Medical 911 calls^g^	Nuisance calls^h^
Treatment × postintervention coefficient (SE)^i^	−0.24 (0.15)	−0.12 (0.09)	−1.21 (0.29)^j^	−1.37 (0.34)^j^	−0.91 (0.22)^j^	−0.17 (0.06)^k^	−0.41 (0.18)^l^	−0.18 (0.24)
Change (95% CI), %^m^	−21.4 (−41.3 to 5.2)	−11.1 (−26.1 to 6.9)	−70.2 (−83.0 to −47.5)	−74.5 (−87.0 to −50.0)	−59.7 (−73.8 to −38.0)	−15.9% (−25.1 to −5.6)	−33.4 (−53.0 to −5.5)	−16.6 (−47.7 to 33.1)
Preintervention crime count, mean (SD)	4.9 (4.8)	2.3 (2.1)	0.8 (1.4)	9.0 (17.0)	2.7 (5.6)	141.1 (105.0)	69.6 (78.5)	31.4 (37.0)
No. of observations	2736	2736	2544	2736	2736	2736	2736	2736

Abbreviation: OPC, overdose prevention center.

^a^ Data are from an analysis that encompassed a tesselated 3-hexagon array surrounding the OPC site.

^b^ Includes murder, robbery, and aggravated and simple assault.

^c^ Includes burglary, theft, and motor vehicle theft.

^d^ Refers to criminal possession of a weapon.

^e^ Refers to the unlawful sale or possession of drugs.

^f^ Refers to calls made to police where there was a possible crime in progress or where one had been committed.

^g^ Includes calls requiring an ambulance or the response of fire department medical personnel.

^h^ Includes 911 calls for trespass and 311 calls about homelessness (assisting a person experiencing homelessness, encampments, and homelessness-related street condition) and disorder (rodents, graffiti, dirty and unsanitary conditions, drug and alcohol activity, public urination, and 311 calls under the New York City Police Department’s jurisdiction [eg, abandoned vehicles and noise complaints]).

^i^ Calculated as difference-in-differences Poisson regression estimates on the association of public safety and the opening of the OPCs. The specifications include hexagon and month-year fixed effects. Robust SEs clustered at the hexagon level in parentheses. The specification follows the equation in the eMethods in Supplement 1, where postintervention is an indicator for whether a given observation occurred after November 30, 2021, when the 2 OPCs were opened to the public. Treatment is an indicator for whether a hexagon contains 1 of the 2 OPCs as opposed to a comparison unit. Hence, Table 3 shows the coefficient on the interaction between postintervention and treat, which is the estimated difference-in-differences intervention effect.

^j^ *P* < .001.

^k^ *P* < .01.

^l^ *P* < .05.

^m^ Calculated as incidence rate ratio − 1 = exp(β) − 1.

#### Calls to 911 and 311

Our analysis did not reveal a significant change in the total number of crime-related 911 calls in the immediate vicinity of the OPCs (Table 2). Estimates for calls about crime complaints were negative (−28.5% [95% CI, −62.3% to 35.6%]), and the trespass estimate was significant (−25.3% [95% CI, −44.2% to 0%]). The estimate for medical calls was likewise negative, but very imprecise (−45.9% [95% CI, −75.2% to 18.1%]). Nuisance calls, an aggregation of 911 calls for trespass complaints and 311 calls for drug activity, noise, abandoned vehicles, and homelessness, did not exhibit a significant change (2.5% [95% CI, −30.1% to 50.3%]).

**Figure 2.**
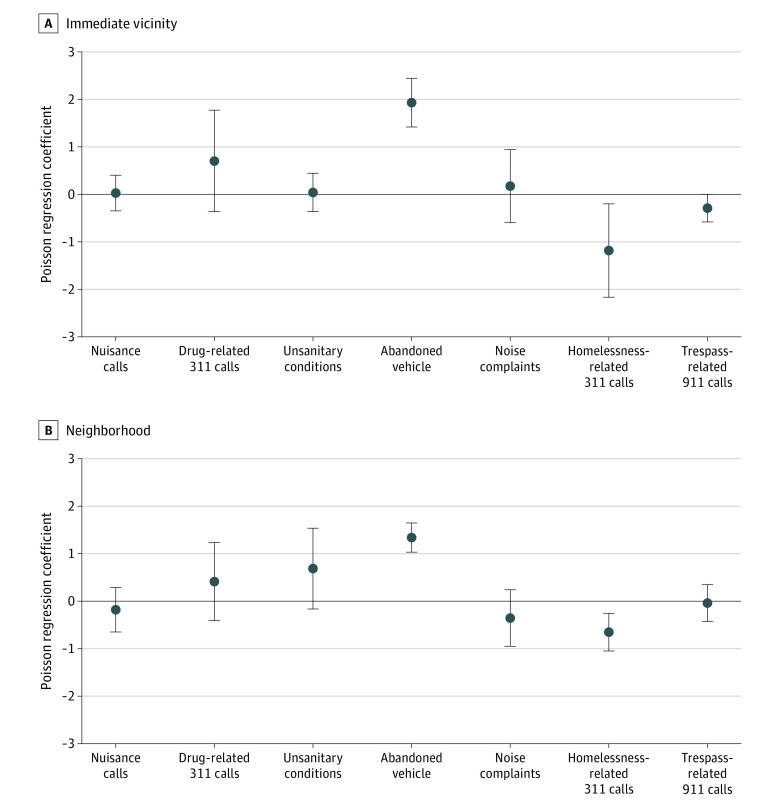
Changes in Frequency of Nuisance Calls After Opening of an Overdose Prevention Center The immediate vicinity of an overdose prevention center (A) is defined as a hexagonal area spanning about 6 city blocks around the site. The wider neighborhood (B) is defined as a tesselated 3-hexagon array spanning about 18 city blocks around the site. Error bars indicate SEs.

Figure 2 provides additional detail on changes in specific types of 311 calls. In both the immediate vicinity of the OPCs and the broader neighborhood, calls for abandoned vehicles rose (591.4% [95% CI, 313.4% to 1056.5%]), while calls about homelessness fell (−69.5% [95% CI, −88.6% to −18.2%]). Drug-related calls did not change significantly (101.9% [95% CI, −31.0% to 491.1%]), though the base rate (0.5 calls per hexagon month) was low, and the estimate is therefore imprecise. While 311 calls for discarded syringes would be of interest in this study, the number of these calls was too low at all locations and times—fewer than 1 per year—to conduct an analysis with confidence.

In the broader neighborhoods around the OPCs (Table 3), crime-related 911 calls fell by 15.9% (95% CI, −25.1% to −5.6%) around the OPCs relative to comparison areas. We also observed a 33.4% decline in 911 calls related to medical conditions (95% CI, −53.0% to −5.5%). We did not observe a significant change in nuisance-related calls (−16.6% [95% CI, −47.7% to 33.1%]).

#### Police Enforcement

After the OPCs opened, relative to SSP comparison sites, arrests for drug and weapons possession in the immediate vicinity of the OPCs decreased by 82.7% (95% CI, −89.9% to −70.4%) and by 56.5% (95% CI, −79.4% to −8.1%), respectively, both significant results. We similarly observed a 70.2% decrease in weapons arrests (95% CI, −83.0% to −47.5%) and a 74.5% decrease in drug arrests (95% CI, −87.0% to −50.0%) in the broader neighborhood around the OPCs. These results are corroborated by an analysis of criminal court summonses issued around the 2 OPCs, which comparatively decreased by 87.9% in their immediate vicinity (95% CI, −91.9% to −81.9%) and by 59.7% in the broader community (95% CI, −73.8% to −38.0%).

### Sensitivity Analyses

We reestimated our models with controls for preintervention trends (eTable 2 in Supplement 1) using negative binomial regression instead of Poisson regression (eTable 3 in Supplement 1) and adding police patrol borough time trends (eTable 4 in Supplement 1). Event study estimates are presented in eFigures 1 to 3 in Supplement 1. eFigure 4 in Supplement 1 presents a falsification test that randomly assigned an intervention indicator variable to 2 of the 17 SSP sites used as comparisons, resulting in 136 intervention-comparison site combinations. The test suggested the significant changes observed were unlikely to be due to chance. Testing for relative differences in key outcomes between intervention and comparison areas before the OPCs were opened, the analyses do not suggest a violation of the common trends assumption required by difference-in-differences designs.

We also report estimates from 2 alternative comparison groups using other hexagons in the city with similar levels of violent crime and drug arrests (eFigures 8 and 9 in Supplement 1). We added an alternative comparison group for hexagons with high levels of drug arrests since the East Harlem OPC site, in particular, had a relatively high number of drug arrests compared with other locations in the city (eFigure 6 in Supplement 1). The trends observed were similar to those of our primary model (eFigure 7 in Supplement 1). Descriptive statistics are presented in eTable 5 in Supplement 1. Regression estimates for comparison areas with high levels of crime are reported in eTable 6 in Supplement 1; estimates for comparison areas with high levels of drug arrests are reported in eTable 7 in Supplement 1. Event study estimates for the 2 alternative designs are reported in eFigures 10 and 11 in Supplement 1. In all cases, point estimates were consistent with those drawn from our primary models. In eFigure 12 in Supplement 1, we plotted estimates from 5 different specifications for each type of 311 call. Estimates were likewise consistent across models.

## Discussion

To our knowledge, this cohort study is the first to measure the crime and nuisance trends associated with the opening of government-sanctioned OPCs in a US city. In accordance with prior findings that SSPs are not associated with increases in crime and disorder^14^ and analyses of OPCs outside of the US,^1,3,31^ we did not observe significant increases in reported crime, disorder complaints, or related 311 and 911 calls. Where estimates were significant, they pointed to modest decreases in crime reports and medical calls.

The comparative reduction in drug possession arrests and criminal court summonses around the OPCs after they opened was notable. In a city where the police department reports to the mayor’s office, these decreases were consistent with the mayor’s pronounced support for the OPCs^32,33^ and the city’s intention to open several additional locations.^13,34^ The decreases may therefore reflect the municipality’s desire to not deter clients from visiting OPCs if they fear arrests for narcotics possession. In effect, the results may indicate a bundled intervention that merits further research: the addition of overdose prevention services and coordination with police.^35^ While our estimates may therefore not isolate the marginal association of overdose prevention services with crime and disorder in the narrowest sense, they suggest NYC was able to successfully operate OPCs without compromising public safety.

It is possible that public scrutiny placed on the nation’s first sanctioned OPCs led staff to be conscientious about monitoring local safety and public order conditions. Given that 36.9% of the OPC’s clients reported being homeless and 75.9% of all clients state they would have used drugs in public or semipublic places in the instances where they had consumed them at the OPCs,^16^ it is plausible that the OPCs absorbed behaviors that would have generated 911 calls, 311 calls, and resulting police activity.

### Limitations

The principal limitations of this study are inherent in the use of police administrative data. Calls to 911 and 311 are unverified reports of a given incident or behavior. Some calls could be unfounded or duplicate reports, or people may not report the incidents they observe at all. This would not affect the results herein if such reporting behaviors were consistent across our study settings, but it is possible that residents concerned by the prospect of OPCs may have been more active in reporting crime and disorder after the centers opened.

Similar concerns prevail about police activity. Police officers have considerable discretion in recording calls for disorder and nonviolent misdemeanors, as well as their classification and enforcement.^36^ The use of discretion regarding illicit drug-related activity observed herein aligns with consistent support for OPCs by NYC government, but it is possible that it extended to other behaviors of interest in this study. However, police have considerably less discretion when a resident makes an allegation (opposed to incidents that officers observe independently), or when the incident is considered a violent or serious crime. Alongside 911 call records, which are not filtered through police discretion, this provides reason to believe the recording of serious crimes was consistent across sites and time.

Finally, we examined the association of the OPCs with administratively recorded crimes, arrests, and calls for service, which may or may not reflect community residents’ perceptions of safety or disorder. Even if OPCs did not bring additional crime to a community, residents may nevertheless experience higher levels of fear or discomfort in their presence. Future research should investigate local attitudes toward OPCs and how they evolve over time.

## Conclusions

Evaluating a politically controversial public health intervention requires assessing effects on a community that go beyond its proximate health outcomes.^22^ More research is required to conclude that the 2 OPCs in NYC will not be associated with localized increases in crime and disorder over a longer span of time. However, objections to their implementation that rest on these concerns are not necessarily supported by our initial observations in this cohort study. Our findings also suggest that a cooperative relationship between police and OPCs can enhance their effectiveness as a lifesaving intervention while minimizing behaviors that would erode public support for such initiatives.

## Notes

Supplement 1. **eMethods.** Statistical Analysis **eTable 1.** Associations Between Opening an Overdose Prevention Center and Crime **eTable 2.** Associations Between Opening an Overdose Prevention Center and Public Safety, Pretrend Controls **eTable 3.** Associations Between Opening an Overdose Prevention Center and Public Safety, Negative Binomial Regression **eTable 4.** Associations Between Opening an Overdose Prevention Center and Public Safety, Patrol Borough Linear Time Trends **eFigure 1.** Poisson Event Study Design Estimates on Public Safety **eFigure 2.** Poisson Event Study Design Estimates on Crime **eFigure 3.** Poisson Event Study Design Estimates on Calls for Service and Criminal Summons **eFigure 4.** Falsification Test on the Association Between Opening an Overdose Prevention Center and Public Safety **eFigure 5.** Public Safety Time Series by Site, Quarter Hexagon Level Data **eTable 5.** Descriptive Statistics by Sample Selection, Hexagon Monthly Level Data **eTable 6.** Association Between Opening an Overdose Prevention Center and Public Safety, High Crime Comparison Sample **eTable 7.** Association Between Opening an Overdose Prevention Center and Public Safety, High Drug Arrests Comparison Sample **eTable 8.** Neighborhood Sociodemographic Characteristics by Intervention Status, 5-Year Estimates **eFigure 6.** Histogram of Preintervention Drug Arrests by Hexagonal Area, New York City **eFigure 7.** Public Safety Time Series by Sample, Bimonthly Hexagon Level Data **eFigure 8.** Spatial Distribution of the High Crime Hexagons Sample **eFigure 9.** Spatial Distribution of the High Drug Arrests Hexagons Sample **eFigure 10.** Poisson Event Study Design Estimates on Public Safety, High Crime Sample **eFigure 11.** Poisson Event Study Design Estimates on Public Safety, High Drug Arrests Sample **eFigure 12.** Difference-in-Differences Estimates on Nuisance Calls by Model Specification

Supplement 2.
Data Sharing Statement
