# Supplementary material for: Overdose Prevention Centers, Crime, and Disorder in New York City
Source: JAMA Netw Open. 2023 Nov 13;6(11):e2342228. doi: 10.1001/jamanetworkopen.2023.42228 (PMC10644216; doi:10.1001/jamanetworkopen.2023.42228)
Supplement: Supplement 1. — eMethods. Statistical Analysis eTable 1. Associations Between Opening an Overdose Prevention Center and Crime eTable 2. Associations Between Opening an Overdose Prevention Center and Public Safety, Pretrend Controls eTable 3. Associations Between Opening an Overdose Prevention Center and Public Safety, Negative Binomial Regression eTable 4. Associations Between Opening an Overdose Prevention Center and Public Safety, Patrol Borough Linear Time Trends eFigure 1. Poisson Event Study Design Estimates on Public Safety eFigure 2. Poisson Event Study Design Estimates on Crime eFigure 3. Poisson Event Study Design Estimates on Calls for Service and Criminal Summons eFigure 4. Falsification Test on the Association Between Opening an Overdose Prevention Center and Public Safety eFigure 5. Public Safety Time Series by Site, Quarter Hexagon Level Data eTable 5. Descriptive Statistics by Sample Selection, Hexagon Monthly Level Data eTable 6. Association Between Opening an Overdose Prevention Center and Public Safety, High Crime Comparison Sample eTable 7. Association Between Opening an Overdose Prevention Center and Public Safety, High Drug Arrests Comparison Sample eTable 8. Neighborhood Sociodemographic Characteristics by Intervention Status, 5-Year Estimates eFigure 6. Histogram of Preintervention Drug Arrests by Hexagonal Area, New York City eFigure 7. Public Safety Time Series by Sample, Bimonthly Hexagon Level Data eFigure 8. Spatial Distribution of the High Crime Hexagons Sample eFigure 9. Spatial Distribution of the High Drug Arrests Hexagons Sample eFigure 10. Poisson Event Study Design Estimates on Public Safety, High Crime Sample eFigure 11. Poisson Event Study Design Estimates on Public Safety, High Drug Arrests Sample eFigure 12. Difference-in-Differences Estimates on Nuisance Calls by Model Specification [file jamanetwopen-e2342228-s001.pdf]

Supplementary Online Content

Chalfin A, del Pozo B, Mitre-Becerril D. Overdose prevention centers, crime, and disorder in New York City. *JAMA Netw Open*. 2023;6(11):e2342228.
doi:10.1001/jamanetworkopen.2023.42228

eMethods. Statistical Analysis
eTable 1. Associations Between Opening an Overdose Prevention Center and Crime
eTable 2. Associations Between Opening an Overdose Prevention Center and Public Safety, Pretrend Controls
eTable 3. Associations Between Opening an Overdose Prevention Center and Public Safety, Negative Binomial Regression
eTable 4. Associations Between Opening an Overdose Prevention Center and Public Safety, Patrol Borough Linear Time Trends
eFigure 1. Poisson Event Study Design Estimates on Public Safety
eFigure 2. Poisson Event Study Design Estimates on Crime
eFigure 3. Poisson Event Study Design Estimates on Calls for Service and Criminal Summons
eFigure 4. Falsification Test on the Association Between Opening an Overdose Prevention Center and Public Safety
eFigure 5. Public Safety Time Series by Site, Quarter Hexagon Level Data
eTable 5. Descriptive Statistics by Sample Selection, Hexagon Monthly Level Data
eTable 6. Association Between Opening an Overdose Prevention Center and Public Safety, High Crime Comparison Sample
eTable 7. Association Between Opening an Overdose Prevention Center and Public Safety, High Drug Arrests Comparison Sample
eTable 8. Neighborhood Sociodemographic Characteristics by Intervention Status, 5-Year Estimates
eFigure 6. Histogram of Preintervention Drug Arrests by Hexagonal Area, New York City
eFigure 7. Public Safety Time Series by Sample, Bimonthly Hexagon Level Data
eFigure 8. Spatial Distribution of the High Crime Hexagons Sample
eFigure 9. Spatial Distribution of the High Drug Arrests Hexagons Sample
eFigure 10. Poisson Event Study Design Estimates on Public Safety, High Crime Sample
eFigure 11. Poisson Event Study Design Estimates on Public Safety, High Drug Arrests Sample
eFigure 12. Difference-in-Differences Estimates on Nuisance Calls by Model Specification

This supplementary material has been provided by the authors to give readers additional information about their work.

© 2023 Chalfin A et al. *JAMA Network Open*.

eMethods. Statistical Analysis

The association of overdose prevention sites with indicators of crime and disorder is estimated using the following regression equation which, for ease of exposition, we represent using ordinary least squares:

$$Y_{it}^{j} = \alpha + \gamma POST_t + \theta TREAT_i + \beta(POST_t * TREAT_i) + \varepsilon_{it}$$

In the equation above, Y^{j}_{it} is the number of crimes known to law enforcement or calls for service of type *j* in each hexagon area, *i*, and month, *t*. $POST_t$ is an indicator for whether a given observation occurs after December, 2021 when the 2 safe injection sites were opened to the public. $TREAT_i$ is an indicator for whether a hexagon contains one of the 2 OPCs as opposed to a comparison α is the constant term measuring the average of the outcome for the comparison group during the pre-intervention period. γ captures the average difference in the outcome from the pre- to post-intervention period common to both groups. θ measures the average difference in the outcome between the intervention and comparison groups common to both pre- and post-intervention periods. β, the coefficient on the interaction between $POST_t$ and $TREAT_i$ is the estimated difference-in-differences intervention association. In practice, given the count distribution of our data, we estimate (1) using Poisson regression, reporting results from a negative binomial regression model in **eTable 3**. In all analyses, standard errors are clustered by hexagon to account for arbitrary heteroskedasticity and spatial dependence.

In order to study whether crime proliferated to the wider neighborhood beyond the immediate vicinity of a site, we re-estimate (1), aggregating the immediate hexagon with 2 adjacent hexagons to form a measure of crime in the broader community (see **Figure 1**, Panel C). Finally, recognizing that differences-in-differences estimates are causally identified under the assumption of parallel trends, we also estimate an event study model in which we report estimated intervention associations in relative time. Specifically, the model uses the following equation:

$$y_{it} = \omega_i + \sigma_t + \sum_{\tau=-q}^{m} \beta_\tau D_{it} + e_{it}$$

Here, coefficients on the lag terms test whether intervention and comparison hexagons were experiencing parallel trends prior to the opening of the 2 OPCs. When all of the lag coefficients are close to zero, this indicates that comparison areas and intervention areas are well matched on both pre-intervention levels and trends. If the lag coefficients are either positive or negative but constant, this indicates that comparison sites have different pre-intervention levels but continue to share common trends. To the extent that the lag coefficients are rising or falling, the assumption of parallel trends may not be met. To account for arbitrary serial correlation and heteroskedasticity, in all models standard errors are clustered by hexagon.

© 2023 Chalfin A et al. *JAMA Network Open*.

Additional results and sensitivity analyses

We tested the sensitivity of our primary estimates using alternative comparison groups and specifications. We began by establishing that there are common pre-intervention trends among the intervention and comparison locations, an analysis that is reported in **eFigure 1**.[1] For each outcome, intervention and comparison locations appear well-matched and there was little evidence of a pre-intervention deviation in trends. A potential exception was drug arrests, where the intervention sites have considerably higher pre-intervention levels of drug arrests than comparison sites. While drug arrests did not change relative to the comparison sites until the month before the intervention, we addressed the potential for the violation of a parallel trends assumption by adding a series of dummy control variables for relative quarters prior to the intervention. These estimates were qualitatively similar to our preferred estimates and are reported in **eTable 2**. We also report estimates from a negative binomial regression in **eTable 3**, confirming that our estimates are not sensitive to functional form assumptions.

To the extent that each area experiences differential crime trends over time and those trends are correlated in time with the opening of the OPCs, confounding could be an issue. To more directly address this possibility, Appendix **eTable 4** includes police patrol borough time trends to account for secular changes affecting specifically each New York City patrol borough (New York City is divided into eight boroughs – Manhattan North, Manhattan South, Queens North, Queens South, Brooklyn North, Brooklyn South, Bronx and Staten Island). The results hold to this alternative specification.

Next, when analyzing multiple outcomes, there is a concern that significant changes may arise even if there are no true association between the intervention and our outcomes of interest. To rule out this concern, we conducted a randomization inference test by randomly assigning an intervention indicator variable to 2 syringe exchange sites out of the 17 sites used as comparisons, resulting in 136 intervention-comparison combinations. **eFigure 4** shows that the decreases in crime, calls for service, and criminal summons were unlikely to be due to chance.

eFigure 5 presents the raw monthly incident counts per site. While drug arrests and crime and medical 911 calls are atypical high relative to the rest of the SSPs, it shows that sites have great variability in their quarterly outcomes, suggesting the need for controlling for seasonal effects and site-specific confounders, which is accounted for by the difference-in-differences specification.

eTable 5 presents descriptive statistics across the entirety of New York City, high-crime areas, areas of high-drug arrests, and the intervention sample. The areas with the OPCs are negatively self-selected, meaning they recorded more crime and nuisance conditions than their counterparts, and New York City overall. The identifying assumption of the difference-in-differences is not similar levels in outcomes, however, but similar pre-trends.

[1] **Appendix Figures 2** and **3** show the event study design estimates for individual crime categories as well as calls for service, and criminal summons.

© 2023 Chalfin A et al. *JAMA Network Open*.

In **eTable 6,** we report estimates from models in which we used an alternative comparison group. Instead of comparing locations containing the OPCs to other NYC locations with SSPs, we instead compared the intervention hexagons to other hexagons in the city drawn from precincts with similar crime and disorder levels. Specifically, this alternative comparison group uses the 250 hexagons with the most index crimes (excluding theft) among the seven (i.e., 10%) of precincts with the highest index crime rates (excluding theft). The resulting estimates were consistent with those drawn from our preferred models. In **eTable 7** we used a second alternative group by selecting the 20 hexagons with the most pre-intervention drug arrests across New York City, to ensure we conducted a comparison with areas with similar drug arrests levels to the OPCs. The estimates are consistent with the main findings.

Finally, **eTable 8** presents population weighted sociodemographic zip code level data. While this geographical level is larger than the neighborhood examined in the models, it provides some idea about the composition of the intervention and comparison areas. Specifically, both have a similar White population (20%), but the OPCs are in areas with fewer Black (16.1% vs 36.3%) and Asian people (3.4% vs 9.4%) and a greater Hispanic population (58.3% vs 30.7%). The differences in income levels are relatively similar in magnitude across both areas, revealing a per capita income level of $36,000 and 22% of the population under the poverty line. Likewise, both neighborhoods have a similar proportion of the population with at least a bachelor's degree (34%) and born abroad (35.9% vs 31.4%). Consistent with the public safety data, the neighborhoods studied in this research are economically disadvantaged relative to the rest of New York City.

© 2023 Chalfin A et al. *JAMA Network Open*.

eTable 1. Associations Between Opening an Overdose Prevention Center and Crime

	Index crime	Violent	Property	Robbery	Assault	Aggr Assault	Simple Assault	Burglary	Theft	Vehicle theft
	(1)	(2)	(3)	(4)	(5)	(6)	(7)	(8)	(9)	(10)
A. Immediate vicin										
Treat*Post	0.02	−0.08	0.02	−0.28	−0.02	0.27**	−0.22	0.10	−0.07	0.48
	(0.11)	(0.11)	(0.11)	(0.23)	(0.08)	(0.08)	(0.16)	(0.45)	(0.12)	(0.37)
Percent change	2.0%	-7.8%	2.1%	-24.5%	-1.9%	30.4%	-19.7%	10.4%	-6.6%	61.5%
95% CI (%)	-17.4, 25.9	-25.8, 14.5	-17.0, 25.6	-51.6, 18.0	-16.2, 14.9	10.4, 54.0	-41.5, 10.2	-54.1, 165.6	-26.1, 18.1	-22.4, 236.1
Mean	6.7	7.7	3.1	1.7	5.9	1.9	4.1	0.6	2.3	0.2
Observations	912	912	912	912	912	912	912	912	912	874
B. Neighborhood										
Treat*Post	−0.17	−0.24	−0.12	−0.43*	−0.20	−0.08	−0.27	−0.15	−0.25*	0.53**
	(0.11)	(0.15)	(0.09)	(0.18)	(0.15)	(0.16)	(0.20)	(0.11)	(0.11)	(0.18)
Percent change	-15.7%	-21.4%	-11.1%	-34.9%	-17.7%	-7.5%	-23.6%	-13.5%	-21.8%	69.1%
95% CI (%)	-31.8, 4.2	-41.3, 5.2	-26.1, 6.9	-54.0, -7.8	-38.1, 9.3	-32.8, 27.4	-48.5, 13.3	-30.6, 7.7	-37.5, -2.1	18.3, 141.7
Mean	4.7	4.9	2.3	1.1	3.8	1.2	2.5	0.5	1.6	0.2
Observations	2,736	2,736	2,736	2,736	2,736	2,736	2,736	2,688	2,736	2,592

Notes: Difference-in-differences Poisson regression estimates on the crime effects of opening the overdose prevention centers. The specifications include hexagon and month-year fixed effects. Robust standard errors clustered at the hexagon level in parentheses. The specification follows the equation in the Supplementary Material: Statistical Methods, where POST is an indicator for whether a given observation occurs after December, 2021 when the 2 safe injection sites were opened to the public. TREAT is an indicator for whether a hexagon contains one of the 2 OPCs as opposed to a comparison unit. Hence, the table shows the coefficient on the interaction between POST and TREAT, which is the estimated difference-in-differences intervention effect. Index crimes include six UCR part I crimes (murder, robbery, aggravated assault, burglary, theft, and motor vehicle). Violent crimes include murder, robbery, and aggravated and simple assault. Property crimes include burglary, theft, and motor vehicle theft. Panel A examines the immediate vicinity (a single hexagon with site at its center). Panel B inspects the neighborhood (three hexagons surrounding the site). The bottom rows exhibit the percentage change (incidence rate ratio - 1 = exp(β)-1), followed by the 95 percent confidence interval, and the pre-intervention mean count crime on the neighborhoods with OPCs and the number of observations. *$p<0.05$; **$p<0.01$; ***$p<0.001$.

© 2023 Chalfin A et al. *JAMA Network Open*.

eTable 2. Associations Between Opening an Overdose Prevention Center and Public Safety, Pretrend Controls

	Crime		Law enforcement			Calls for service		
	Violent	Property	Weapons arrests	Drug arrests	Criminal summons	Crime 911 calls	Medical 911 calls	Nuisance calls
	(1)	(2)	(3)	(4)	(5)	(6)	(7)	(8)
A. Immediate vicin								
Treat*Post	−0.20	−0.10	−0.86	−1.77***	−2.16***	−0.38	−0.62	0.09
	(0.16)	(0.11)	(0.44)	(0.33)	(0.24)	(0.34)	(0.41)	(0.16)
Percent change	-17.9%	-9.7%	-57.7%	-83.0%	-88.5%	-31.8%	-46.2%	9.8%
95% CI	-40.5%, 13.2%	-27.1%, 11.9%	-82.2%, 0.5%	-91.0%, -67.7%	-92.8%, -81.6%	-64.9%, 32.6%	-76.0%, 20.5%	-19.8%, 50.3%
Mean	7.7	3.1	1.0	17.9	4.9	152.0	90.4	26.0
Observations	912	912	912	912	912	912	912	912
B. Neighborhood								
Treat*Post	−0.29	−0.21*	−1.24***	−1.35***	−0.86***	−0.19***	−0.43*	−0.18
	(0.18)	(0.09)	(0.29)	(0.34)	(0.24)	(0.06)	(0.18)	(0.21)
Percent change	-24.9%	-19.1%	-71.0%	-74.0%	-57.5%	-17.6%	-34.7%	-16.7%
95% CI	-46.9%, 6.1%	-32.5%, -2.9%	-83.6%, -48.9%	-86.7%, -49.0%	-73.6%, -31.5%	-26.4%, -7.7%	-54.3%, -6.7%	-44.9%, 25.8%
Mean	4.9	2.3	0.8	9.0	2.7	141.1	69.6	31.4
Observations	2,736	2,736	2,544	2,736	2,736	2,736	2,736	2,736

Notes: Difference-in-differences Poisson regression estimates on the association of public safety and the opening of the overdose prevention centers. The specifications include hexagon and month-year fixed effects and controls for two four-month bin pre-intervention indicator variables as follows: $y_{it} = \omega_i + \sigma_t + \sum_{\tau=-2}^{-3} \beta_{0\tau} D_{it} + \beta_1 D_{it} + e_{it}$. Robust standard errors clustered at the hexagon level in parentheses. POST is an indicator for whether a given observation occurs after December, 2021 when the 2 safe injection sites were opened to the public. TREAT is an indicator for whether a hexagon contains one of the 2 OPCs as opposed to a comparison unit. Hence, the table shows the coefficient on the interaction between POST and TREAT, which is the estimated difference-in-differences intervention effect. Violent crimes include murder, robbery, and aggravated and simple assault. Property crimes include burglary, theft, and motor vehicle theft. Weapons refer to criminal possession of a weapon. Drugs mean the unlawful sale or possession of drugs. Crime 911 calls refer to those made to police where there was a possible crime in progress or one had been committed. Medical calls include those requiring an ambulance or the response of fire department medical personnel. Nuisance calls include 911 calls for trespass and 311 calls about homelessness (assisting a homeless person, encampment, and homeless street condition) and disorder (rodents, graffiti, dirty and unsanitary conditions, drug and drinking activity, urinating in public, and those 311 calls under the New York Police Department's jurisdiction such as abandoned vehicles and noise complaints). Panel A examines the immediate vicinity (a single hexagon with the site at its center). Panel B inspects the neighborhood (three hexagons surrounding the site). The bottom rows exhibit the percentage change (incidence rate ratio - 1 = exp(β)-1), followed by the 95% confidence interval, and the pre-intervention mean count crime on the neighborhoods with OPCs and the number of observations. *p<0.05; **p<0.01; ***p<0.001.

© 2023 Chalfin A et al. *JAMA Network Open*.

eTable 3. Associations Between Opening an Overdose Prevention Center and Public Safety, Negative Binomial Regression

	Crime		Law enforcement			Calls for service		
	Violent	Property	Weapons arrests	Drug arrests	Criminal summons	Crime 911 calls	Medical 911 calls	Nuisance calls
	(1)	(2)	(3)	(4)	(5)	(6)	(7)	(8)
A. Immediate vicin								
Treat*Post	−0.04	0.02	−0.67	−1.36***	−1.74***	−0.16	−0.30	0.04
	(0.13)	(0.10)	(0.47)	(0.37)	(0.23)	(0.36)	(0.47)	(0.13)
Percent change	-3.9%	2.4%	-49.0%	-74.4%	-82.5%	-15.1%	-25.6%	3.6%
95% CI	-25.7%, 24.2%	-16.7%, 25.8%	-79.5%, 27.0%	-87.5%, -47.5%	-88.8%, -72.6%	-58.1%, 71.8%	-70.1%, 85.2%	-20.1%, 34.4%
Mean	7.7	3.1	1.0	17.9	4.9	152.0	90.4	26.0
Observations	912	912	912	912	912	912	912	912
B. Neighborhood								
Treat*Post	−0.16	−0.09	−1.18***	−0.81**	−0.70***	−0.14*	−0.21	0.01
	(0.14)	(0.09)	(0.32)	(0.27)	(0.20)	(0.06)	(0.16)	(0.14)
Percent change	-15.0%	-9.0%	-69.1%	-55.3%	-50.4%	-13.1%	-19.2%	1.4%
95% CI	-35.2%, 11.5%	-23.0%, 7.6%	-83.5%, -42.2%	-73.6%, -24.5%	-66.3%, -27.2%	-22.0%, -3.2%	-41.5%, 11.5%	-22.5%, 32.8%
Mean	4.9	2.3	0.8	9.0	2.7	141.1	69.6	31.4
Observations	2,736	2,736	2,544	2,736	2,736	2,736	2,736	2,736

Notes: Difference-in-differences Negative binomial regression estimates on the association of public safety and the opening of the overdose prevention centers. The specifications include hexagon and month-year fixed effects. Robust standard errors clustered at the hexagon level in parentheses. The specification follows the equation in the Supplementary Material: Statistical Methods, where POST is an indicator for whether a given observation occurs after December, 2021 when the 2 safe injection sites were opened to the public. TREAT is an indicator for whether a hexagon contains one of the 2 OPCs as opposed to a comparison unit. Hence, the table shows the coefficient on the interaction between POST and TREAT, which is the estimated difference-in-differences intervention effect. Violent crimes include murder, robbery, and aggravated and simple assault. Property crimes include burglary, theft, and motor vehicle theft. Weapons refer to criminal possession of a weapon. Drugs mean the unlawful sale or possession of drugs. Crime 911 calls refer to those made to police where there was a possible crime in progress or one had been committed. Medical calls include those requiring an ambulance or the response of fire department medical personnel. Nuisance calls include 911 calls for trespass and 311 calls about homelessness (assisting a homeless person, encampment, and homeless street condition) and disorder (rodents, graffiti, dirty and unsanitary conditions, drug and drinking activity, urinating in public, and those 311 calls under the New York Police Department's jurisdiction such as an abandoned vehicle and noise complaints). Panel A examines the immediate vicinity (a single hexagon with the site at its center). Panel B inspects the neighborhood (three hexagons surrounding the site). The bottom rows exhibit the percentage change (incidence rate ratio - 1 = exp(β)-1), followed by the 95% confidence interval, and the pre-intervention mean count crime on the neighborhoods with OPCs and the number of observations. *p<0.05; **p<0.01; ***p<0.001.

© 2023 Chalfin A et al. *JAMA Network Open*.

eTable 4. Associations Between Opening an Overdose Prevention Center and Public Safety, Patrol Borough Linear Time Trends

	Crime		Law enforcement			Calls for service		
	Violent	Property	Weapons arrests	Drug arrests	Criminal summons	Crime 911 calls	Medical 911 calls	Nuisance calls
	(1)	(2)	(3)	(4)	(5)	(6)	(7)	(8)
A. Immediate vicin								
Treat*Post	0.07	0.03	−0.51	−1.84***	−1.80***	−0.34	−0.83*	−0.11
	(0.13)	(0.06)	(0.44)	(0.27)	(0.15)	(0.30)	(0.36)	(0.27)
Percent change	7.5%	2.9%	-40.2%	-84.1%	-83.4%	-29.0%	-56.2%	-10.6%
95% CI	-16.1%, 37.7%	-8.3%, 15.3%	-74.5%, 40.3%	-90.6%, -73.0%	-87.7%, -77.7%	-60.4%, 27.1%	-78.5%, -10.9%	-47.7%, 53.1%
Mean	7.7	3.1	1.0	17.9	4.9	152.0	90.4	26.0
Observations	912	912	912	912	912	912	912	912
B. Neighborhood								
Treat*Post	−0.07	−0.11	−0.77*	−1.49***	−0.97***	−0.20**	−0.46**	−0.29
	(0.11)	(0.09)	(0.31)	(0.24)	(0.22)	(0.06)	(0.17)	(0.33)
Percent change	-7.0%	-10.3%	-53.5%	-77.4%	-62.3%	-18.1%	-37.1%	-25.1%
95% CI	-25.5%, 16.1%	-25.3%, 7.7%	-74.7%, -14.6%	-85.7%, -64.2%	-75.5%, -41.9%	-27.7%, -7.4%	-54.9%, -12.3%	-60.4%, 41.8%
Mean	4.9	2.3	0.8	9.0	2.7	141.1	69.6	31.4
Observations	2,736	2,736	2,544	2,736	2,736	2,736	2,736	2,736

Notes: Difference-in-differences Poisson regression estimates on the association of public safety and the opening of the overdose prevention centers. The specifications include hexagon and month-year fixed effects as well as police patrol borough linear time trends (New York City is divided into eight police patrol boroughs: Manhattan North, Manhattan South, Queens North, Queens South, Brooklyn North, Brooklyn South, Bronx and Staten Island). Robust standard errors clustered at the hexagon level in parentheses. POST is an indicator for whether a given observation occurs after December, 2021 when the 2 safe injection sites were opened to the public. TREAT is an indicator for whether a hexagon contains one of the 2 OPCs as opposed to a comparison unit. Hence, the table shows the coefficient on the interaction between POST and TREAT, which is the estimated difference-in-differences intervention effect. Violent crimes include murder, robbery, and aggravated and simple assault. Property crimes include burglary, theft, and motor vehicle theft. Weapons refer to criminal possession of a weapon. Drugs mean the unlawful sale or possession of drugs. Crime 911 calls refer to those made to police where there was a possible crime in progress or one had been committed. Medical calls include those requiring an ambulance or the response of fire department medical personnel. Nuisance calls include 911 calls for trespass and 311 calls about homelessness (assisting a homeless person, encampment, and homeless street condition) and disorder (rodents, graffiti, dirty and unsanitary conditions, drug and drinking activity, urinating in public, and those 311 calls under the New York Police Department's jurisdiction such as an abandoned vehicles and noise complaints). Panel A examines the immediate vicinity (a single hexagon with the site at its center). Panel B inspects the neighborhood (three hexagons surrounding the site). The bottom rows exhibit the percentage change (incidence rate ratio - 1 = $\exp(\beta)$-1), followed by the 95% confidence interval, and the pre-intervention mean count crime on the neighborhoods with OPCs and the number of observations. *p<0.05; **p<0.01; ***p<0.001.

© 2023 Chalfin A et al. *JAMA Network Open*.

eFigure 1. Poisson Event Study Design Estimates on Public Safety

I. Immediate vicinity

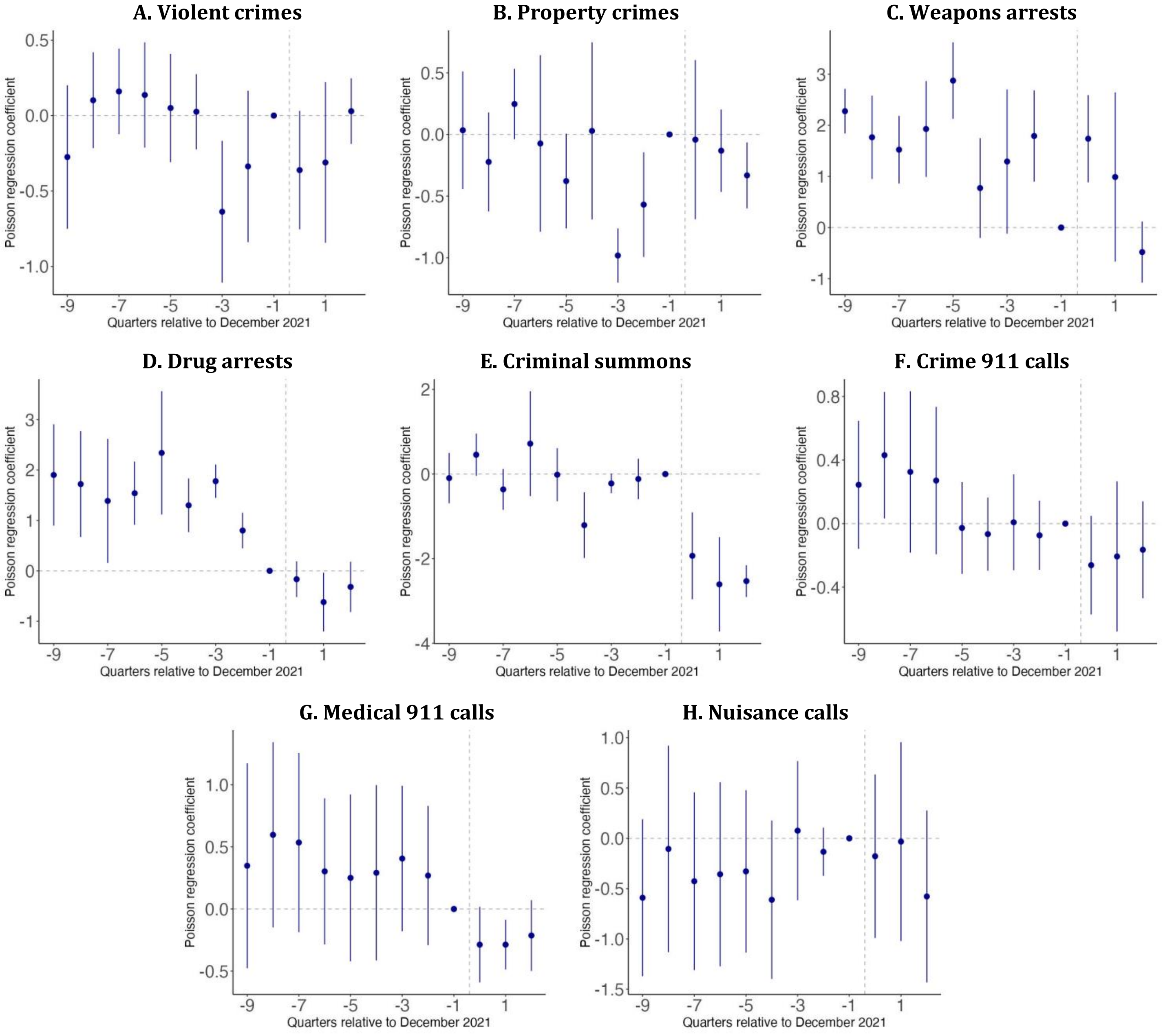

© 2023 Chalfin A et al. *JAMA Network Open*.

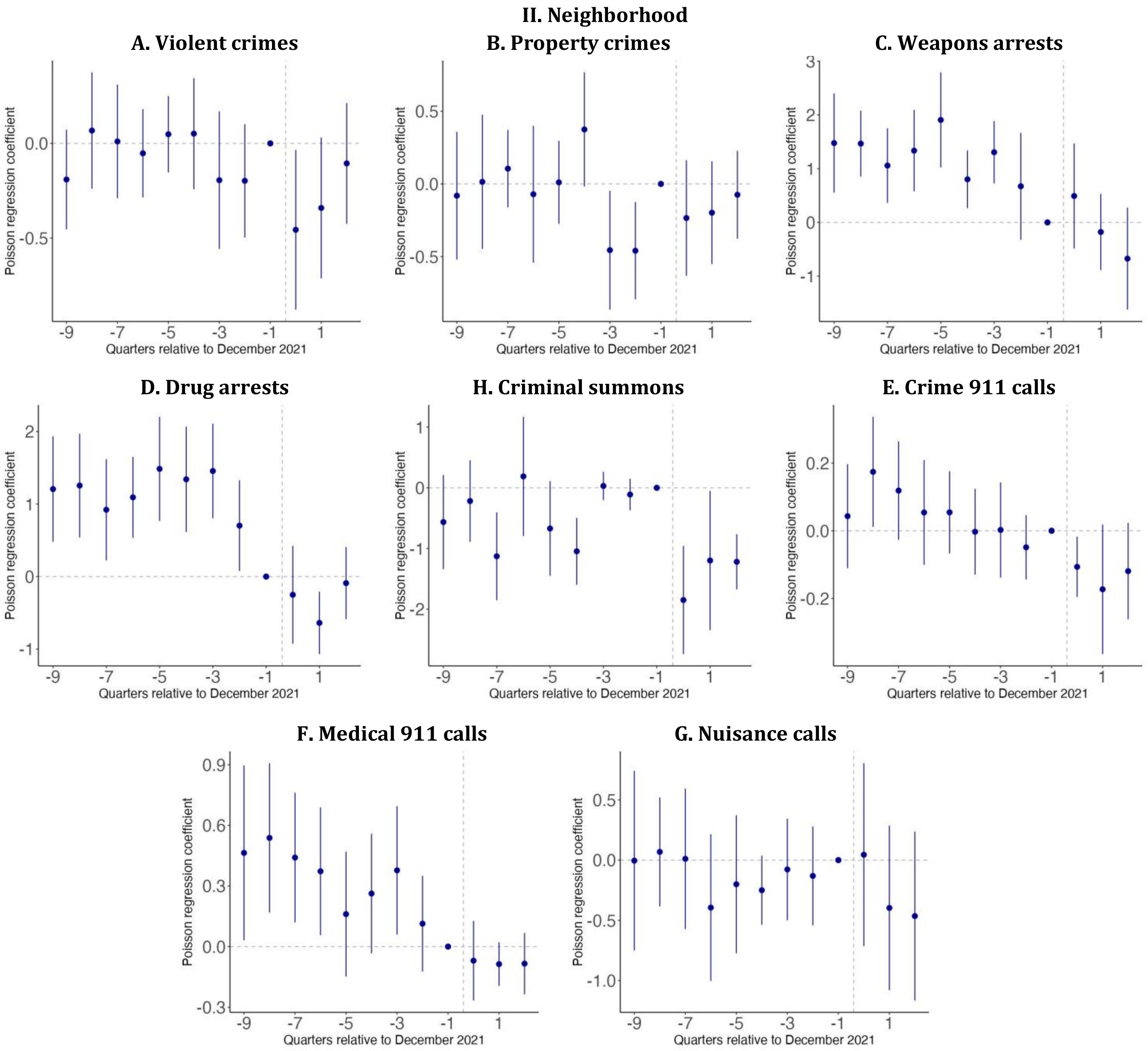

Note: The panels show the event study design estimates using a Poisson regression. Each estimate represents a four-month bin relative to the opening of the overdose prevention centers (December 2021). The models include hexagon and month-year fixed effects. The standard errors are clustered at the hexagon level using the following specification: $y_{it} = \omega_i + \sigma_t + \sum_{\tau=-q}^{m} \beta_\tau D_{it} + e_{it}$. Violent crimes include murder, robbery, and aggravated and simple assault. Property crimes include burglary, theft, and motor vehicle theft. Weapons arrests refer to criminal possession of a weapon. Drugs mean the unlawful sale or possession of drugs. Crime 911 calls refer to those made to police where there was a possible crime in progress or one had been committed. Medical calls include those requiring an ambulance or the response of fire department medical personnel. Nuisance calls include 911 calls for trespass and 311 calls about homelessness (assisting a homeless person, encampment, and homeless street condition) and disorder (rodents, graffiti, dirty and unsanitary conditions, drug and drinking activity, urinating in public, and those 311 calls under the New York Police Department's jurisdiction such as an abandoned vehicle and noise complaints).

© 2023 Chalfin A et al. *JAMA Network Open*.

eFigure 2. Poisson Event Study Design Estimates on Crime

I. Immediate vicinity

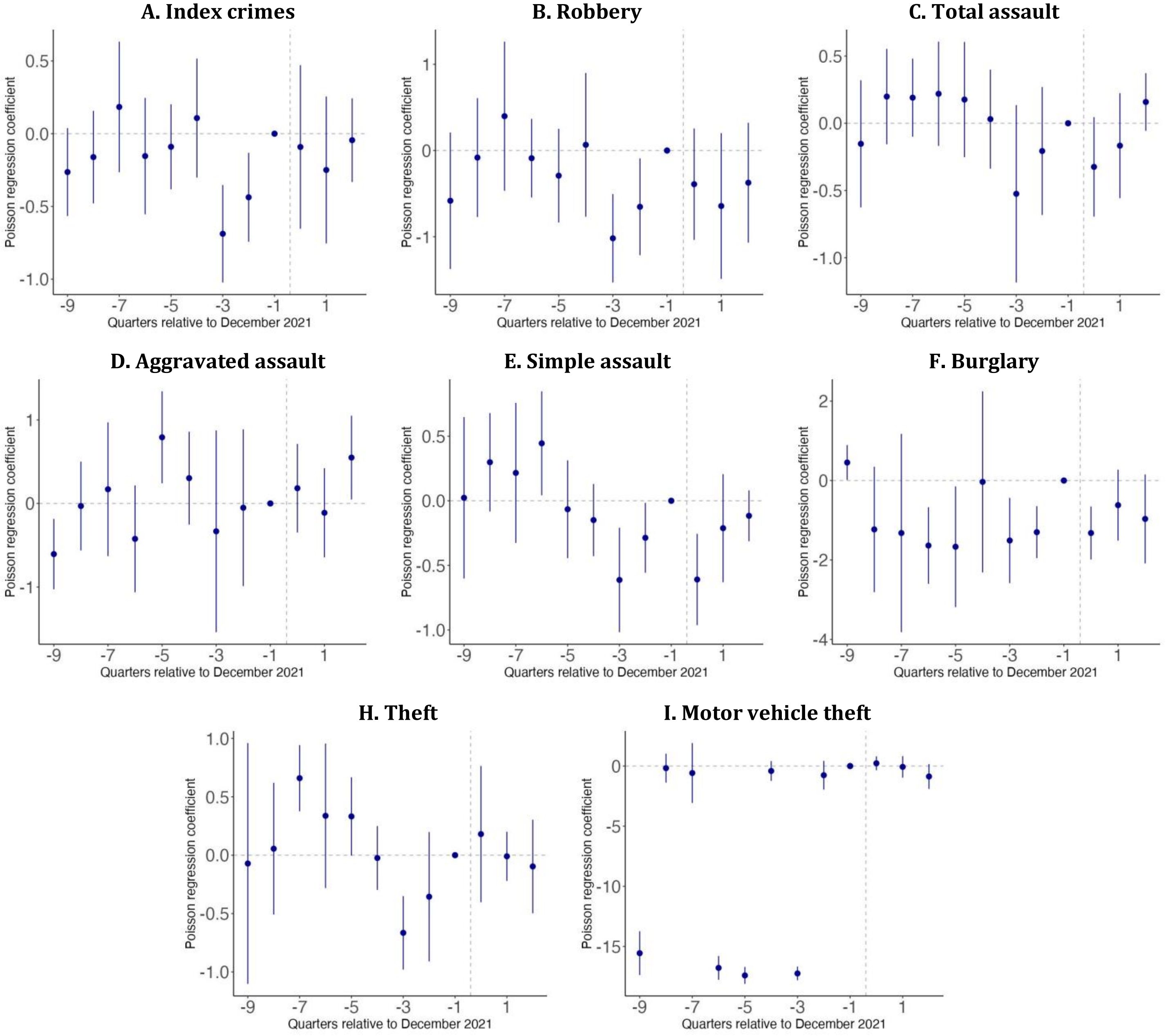

© 2023 Chalfin A et al. *JAMA Network Open*.

II. Neighborhood

A. Index crimes B. Robbery C. Total assault

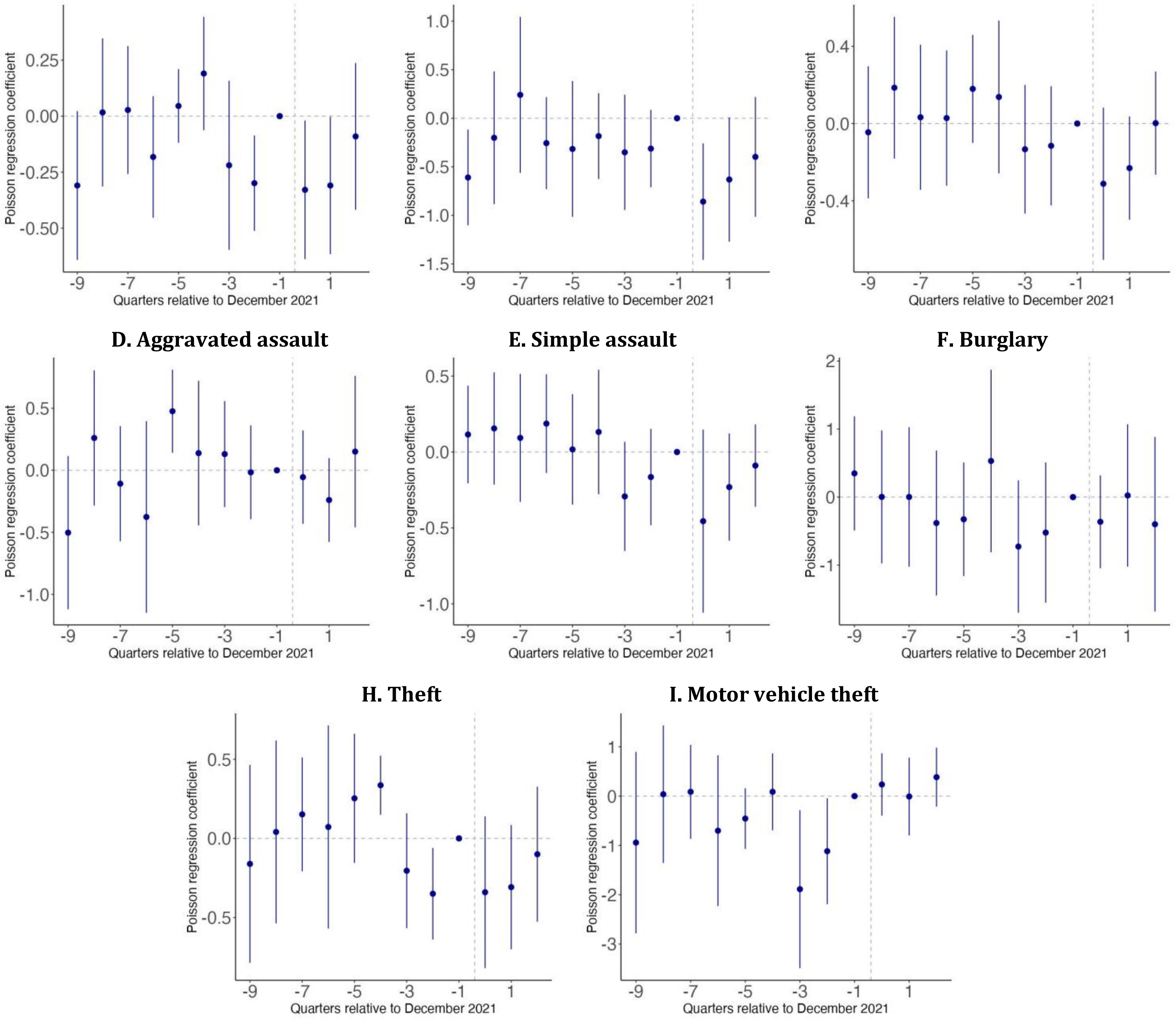

Note: The panels show the event study design estimates using a Poisson regression. Each estimate represents a four-month bin relative to the opening of the overdose prevention centers (December 2021). The models include hexagon and month-year fixed effects. The standard errors are clustered at the hexagon level using the following specification: $y_{it} = \omega_i + \sigma_t + \sum_{\tau=-q}^{m} \beta_\tau D_{it} + e_{it}$.

© 2023 Chalfin A et al. *JAMA Network Open*.

eFigure 3. Poisson Event Study Design Estimates on Calls for Service and Criminal Summons

I. Immediate vicinity

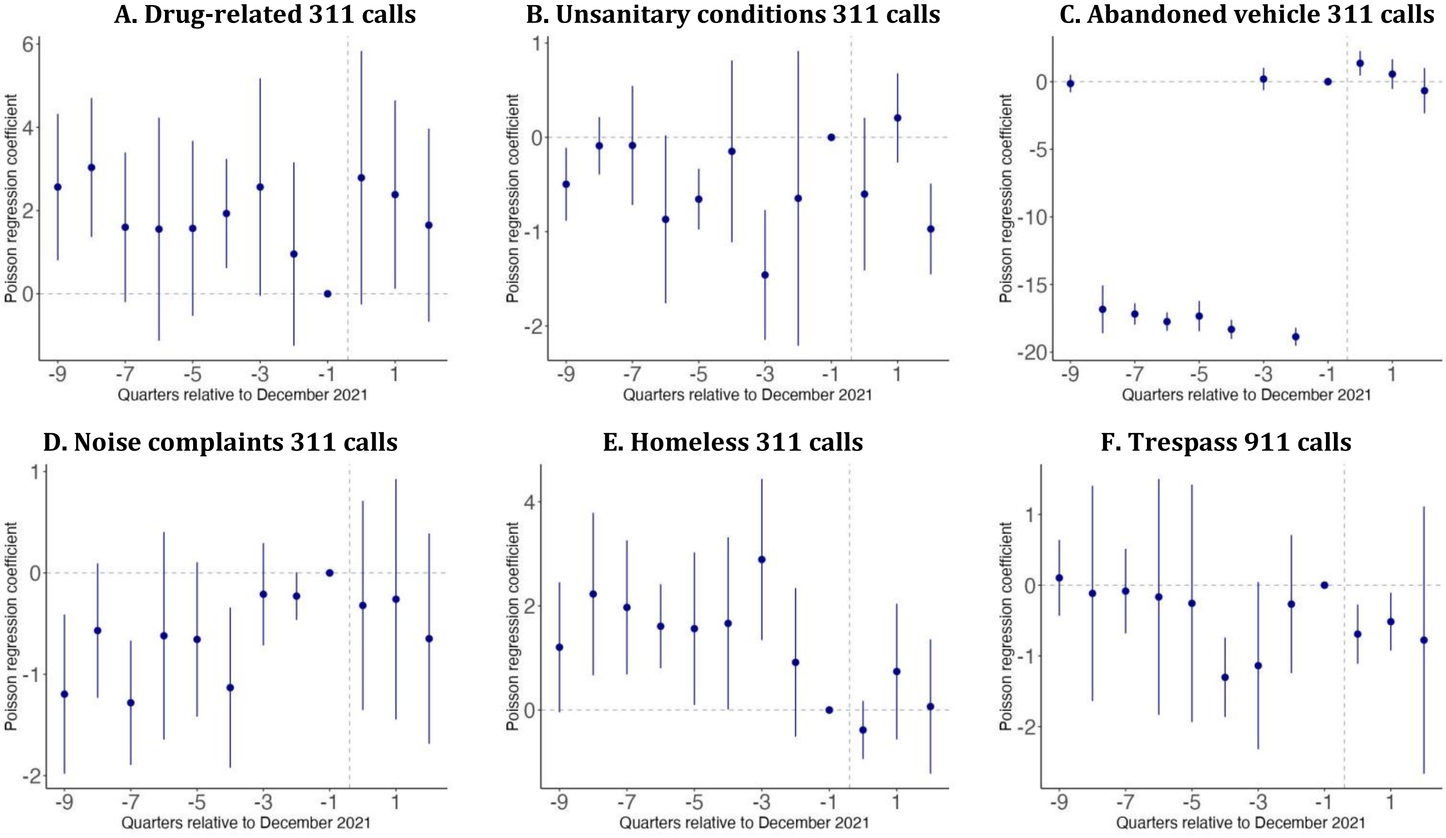

© 2023 Chalfin A et al. *JAMA Network Open*.

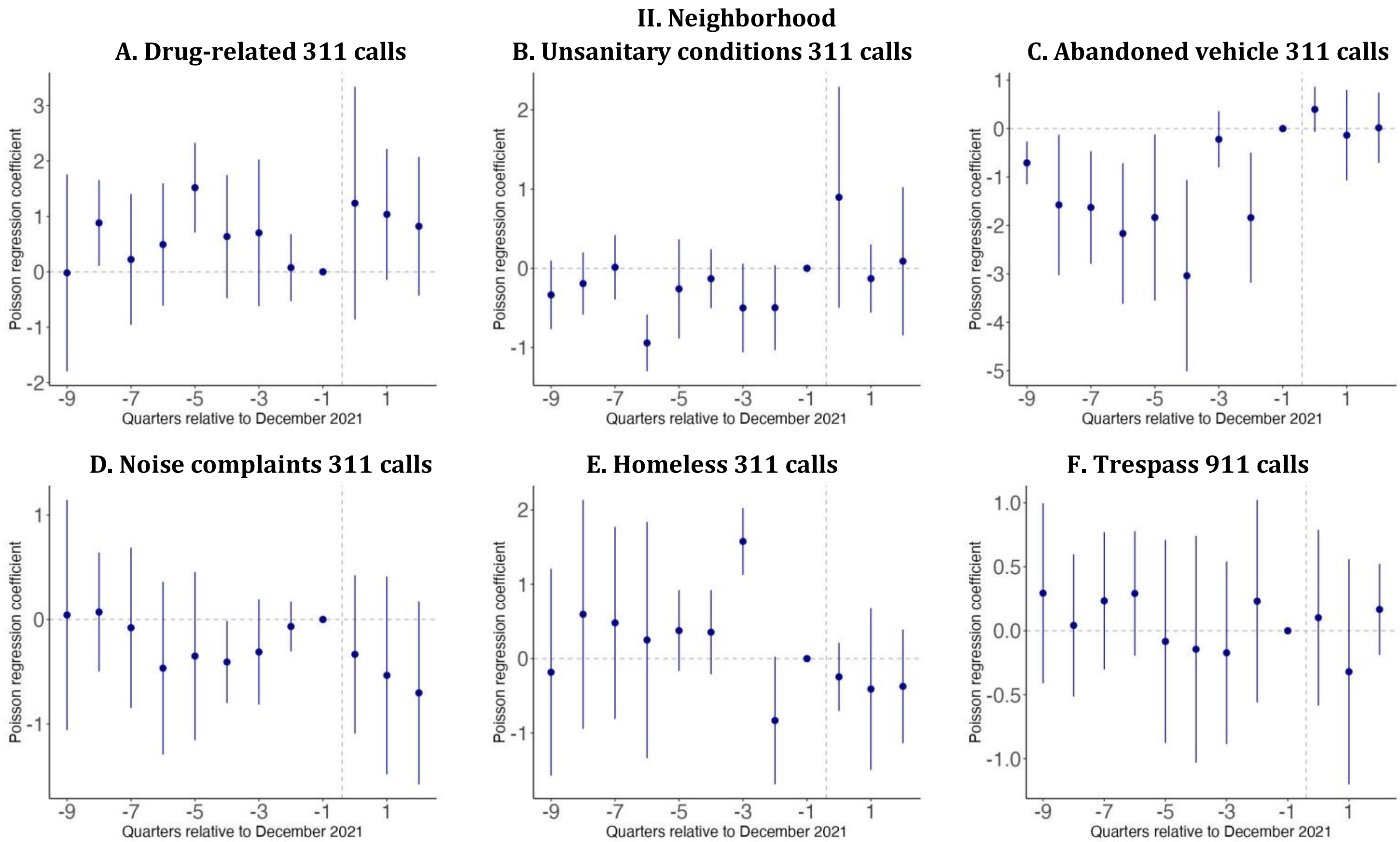

Note: The panels show the event study design estimates using a Poisson regression. Each estimate represents a four-month bin relative to the opening of the overdose prevention centers (December 2021). The models include hexagon and month-year fixed effects. The standard errors are clustered at the hexagon level using the following specification: $y_{it} = \omega_i + \sigma_t + \sum_{\tau=-q}^{m} \beta_\tau D_{it} + e_{it}$. Drug-related 311 calls refer to drug and drinking activity and loose syringes. Unsanitary conditions comprise calls about seeing a rodent, graffiti, dirty and unsanitary conditions, and urinating in public. Abandoned vehicle and noise complaints are calls handled by the New York Police Department. Homeless calls include those related to assisting a homeless person, encampment, and homeless street condition. Trespass 911 calls explicitly mention this offense in the call.

© 2023 Chalfin A et al. *JAMA Network Open*.

eFigure 4. Falsification Test on the Association Between Opening an Overdose Prevention Center and Public Safety

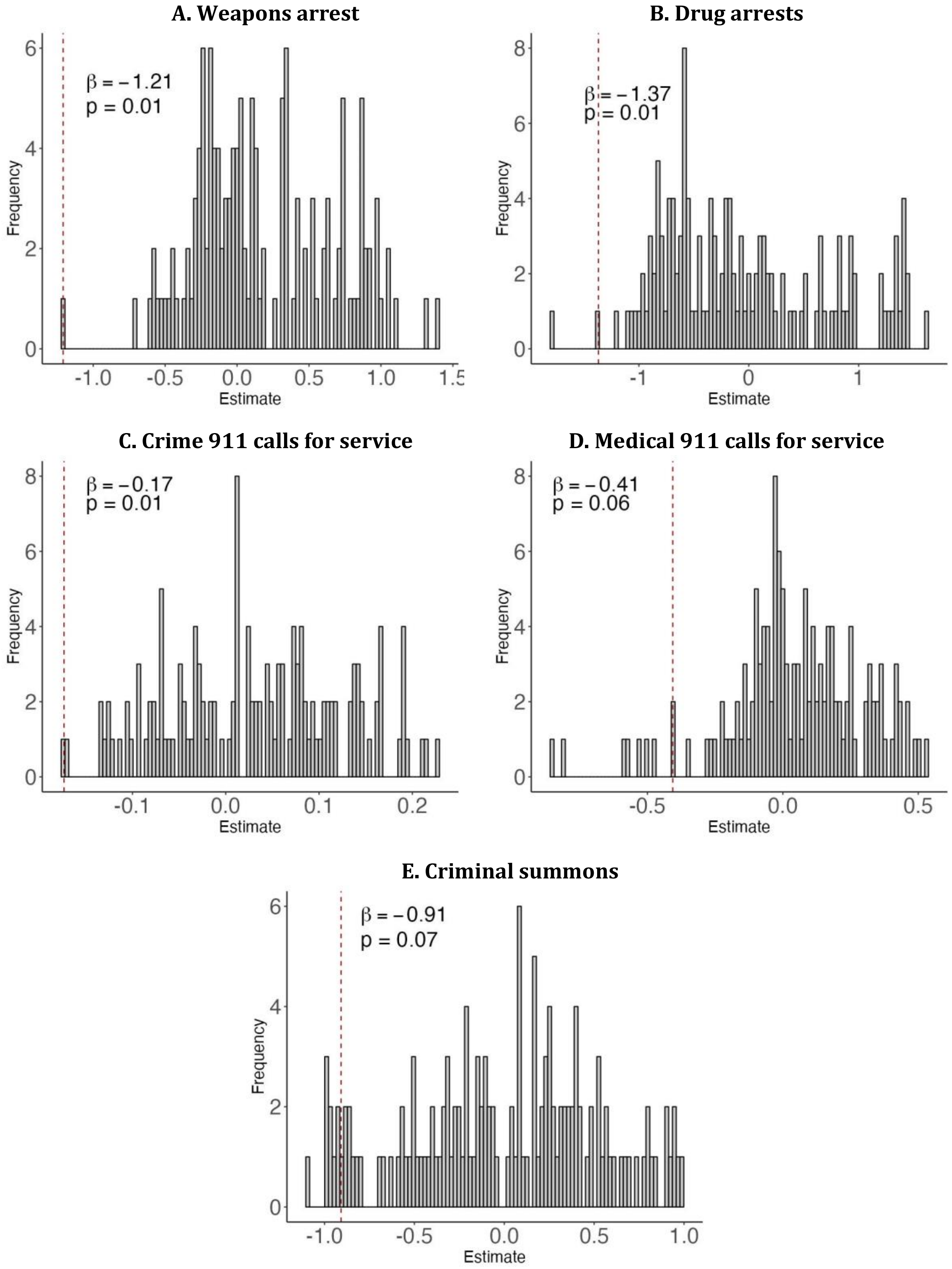

Note: The falsification test randomly assigns an intervention indicator variable to two syringe service programs out of the 17 sites used as comparison. This process results in 136 intervention-comparison combinations. The figures show the difference-in-differences Poisson regression estimates, including hexagon and month-year fixed effects on the neighborhood (three hexagons) sample. Weapons refer to criminal possession of a weapon. Drugs mean the sale or possession of dangerous drugs. Crime 911 calls refer to those made to law enforcement where there was a possible crime in-progress or one has been committed. Medical calls include those needing an ambulance. The vertical lines show the main estimates, along with the implied p-values (rank of the actual intervention association among the placebo intervention association distribution).

© 2023 Chalfin A et al. *JAMA Network Open*.

eFigure 5. Public Safety Time Series by Site, Quarter Hexagon Level Data

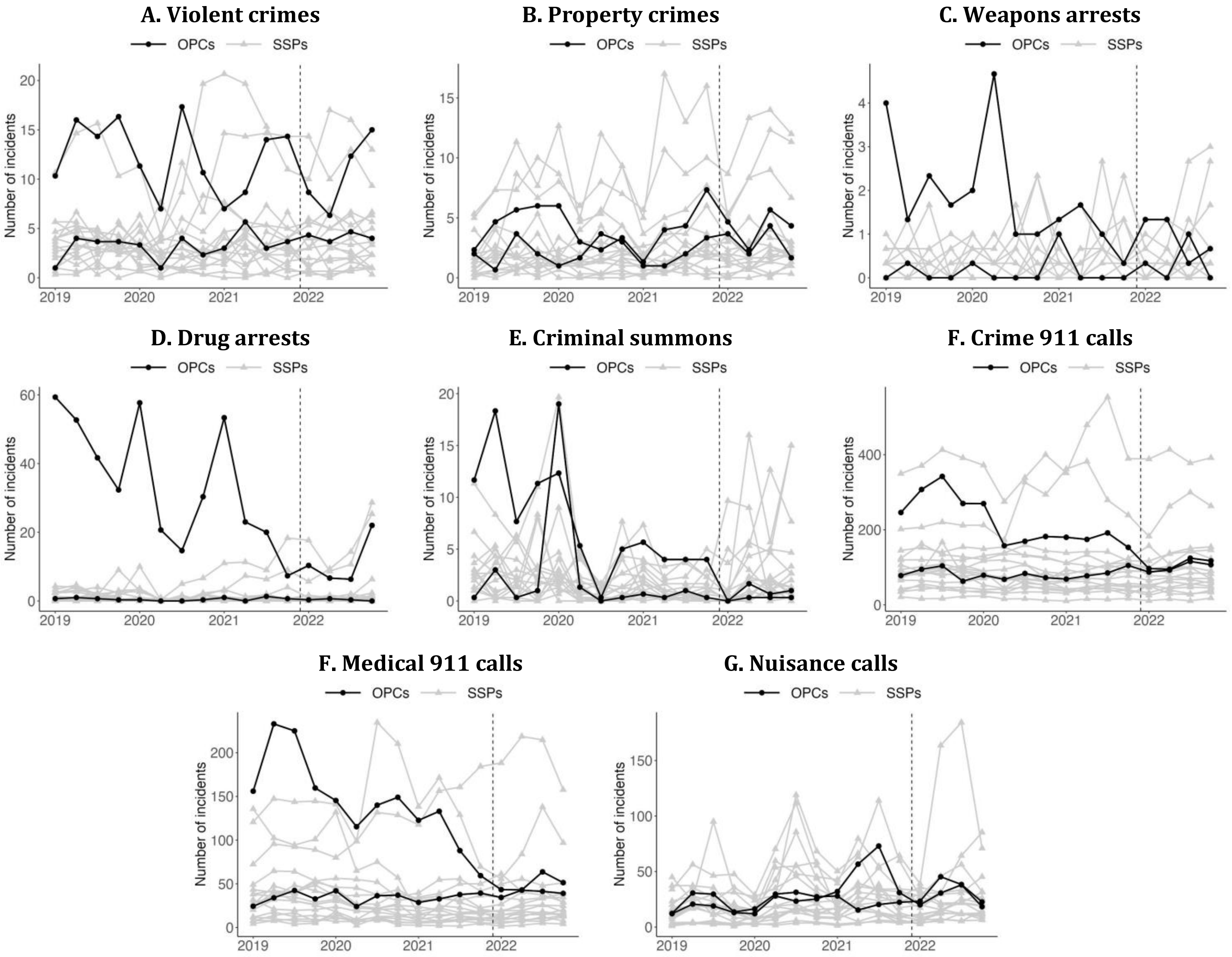

Note: The panels show the number of incidents per hexagon quarter-year by site. Each line represents one of the 19 sites used in the analysis distinguished by OPCS and SSPs. The hexagon surrounds the overdose prevention center. The vertical line marks December 2021, the start of the intervention period. Violent crimes include murder, robbery, and aggravated and simple assault. Property crimes include burglary, theft, and motor vehicle theft. Weapons arrests refer to criminal possession of a weapon. Drugs mean the unlawful sale or possession of drugs. Crime 911 calls refer to those made to police where there was a possible crime in progress or one had been committed. Medical calls include those requiring an ambulance or the response of fire department medical personnel. Nuisance calls include 911 calls for trespass and 311 calls about homelessness (assisting a homeless person, encampment, and homeless street condition) and disorder (rodents, graffiti, dirty and unsanitary conditions, drug and drinking activity, urinating in public, and those 311 calls under the New York Police Department's jurisdiction such as abandoned vehicles and noise complaints).

© 2023 Chalfin A et al. *JAMA Network Open*.

eTable 5. Descriptive Statistics by Sample Selection, Hexagon Monthly Level Data

	Mean count (std. dev.)			
	New York City (n = 8,752)	High-crime neighborhoods (n = 250)	High-drug arrests neighborhoods (n = 20)	Neighborhoods with OPCs (n = 2)
Crimes				
Index	0.9 (1.8)	3.3 (2.5)	5.9 (4.2)	6.7 (4.5)
Violent	0.8 (1.7)	3.7 (2.9)	6.2 (4.2)	7.7 (5.6)
Murder	0.004 (0.1)	0.02 (0.2)	0.02 (0.1)	0.1 (0.3)
Robbery	0.1 (0.4)	0.6 (0.9)	1.2 (1.3)	1.7 (1.8)
Aggravated assault	0.2 (0.6)	1.0 (1.3)	1.7 (1.7)	1.9 (2.1)
Simple Assault	0.4 (1.1)	2.0 (1.9)	3.3 (2.6)	4.1 (3.4)
Property	0.6 (1.3)	1.6 (1.6)	3.0 (2.7)	3.1 (2.3)
Burglary	0.1 (0.4)	0.4 (0.7)	0.6 (1.2)	0.6 (1.0)
Theft	0.4 (1.1)	0.9 (1.3)	2.1 (2.2)	2.3 (2.0)
Motor vehicle theft	0.1 (0.3)	0.3 (0.5)	0.3 (0.6)	0.2 (0.5)
Law enforcement				
Weapons arrests	0.1 (0.4)	0.4 (0.9)	0.6 (1.2)	1.0 (1.5)
Drug arrests	0.1 (0.8)	0.8 (2.8)	6.5 (8.8)	17.9 (22.6)
Criminal summons	0.6 (5.4)	2.2 (6.8)	4.1 (5.5)	4.9 (8.0)
911 calls				
Crime	21.1 (35.4)	79.6 (51.3)	143.1 (76.7)	152.0 (85.2)
Assault	1.2 (2.7)	5.7 (4.4)	8.6 (5.3)	10.3 (7.0)
Trespass	0.2 (0.8)	1.0 (1.9)	1.9 (2.7)	2.2 (1.8)
Medical	6.7 (13.3)	26.8 (21.0)	64.5 (46.4)	90.4 (66.0)
311 calls				
Drug	0.1 (0.6)	0.2 (1.1)	0.5 (1.0)	0.5 (0.9)
Unsanitary conditions	1.1 (2.3)	3.0 (3.5)	3.7 (3.9)	3.1 (3.3)
Abandoned vehicle	0.3 (1.2)	0.4 (1.0)	0.2 (0.6)	0.1 (0.4)
Noise complaint	5.6 (63.3)	15.8 (23.6)	23.9 (38.2)	16.1 (17.0)
Homeless	0.4 (2.6)	0.5 (2.1)	3.4 (8.0)	4.1 (5.3)

Notes: Hexagon monthly-year level pre-intervention (2019M1-2021M11) mean count and standard deviation from all New York City, the high-crime sample, and the intervention hexagon. The New York City sample includes all areas across the city. The high-crime sample was built by first identifying the top seven (10areas that had the highest number of index crimes (excluding larceny) per 100,000 residents, then, selecting the 250 hexagons with the highest volume of crimes among these seven precincts. The high drug arrests sample uses the 20 hexagons with the most pre-intervention mean drug arrests excluding areas in the police precincts where the overdose prevention centers are located. The intervention hexagon surrounds the overdose prevention center. Index crimes include the six UCR part I crimes (murder, robbery, aggravated assault, burglary, theft, and motor vehicle). Violent crimes include murder, robbery, and aggravated and simple assault. Property crimes include burglary, theft, and motor vehicle theft. Weapons possession arrests refer to criminal possession of a weapon, Drug possession arrests mean sale or possession of dangerous drugs. Crime 911 calls include those in which there was a possible crime in-progress or one has been committed. Assault and trespass 911 calls explicitly mention these offenses in the call. Medical calls include those needing an ambulance. Drug related calls include drug and drinking activity and loose syringe calls. Unsanitary conditions comprise calls related to seeing a rodent, graffiti, dirty and unsanitary conditions, and urinating in public. Abandoned vehicle and noise complaints are calls handled by the New York Police Department. Homeless calls include those related to assisting a homeless person, encampment, and homeless street condition.

© 2023 Chalfin A et al. *JAMA Network Open*.

eTable 6. Association Between Opening an Overdose Prevention Center and Public Safety, High Crime Comparison Sample

	Crime		Law enforcement			Calls for service		
	Violent	Property	Weapons arrests	Drug arrests	Criminal summons	Crime 911 calls	Medical 911 calls	Nuisance calls
	(1)	(2)	(3)	(4)	(5)	(6)	(7)	(8)
A. Immediate vicin								
Treat*Post	−0.15	−0.01	−0.59*	−0.81***	−2.03***	−0.36	−0.60	0.09
	(0.08)	(0.05)	(0.29)	(0.13)	(0.10)	(0.31)	(0.35)	(0.14)
Percent change	-14.2%	-0.8%	-44.5%	-55.6%	-86.9%	-30.3%	-45.1%	9.9%
95% CI	-27.1%, 1.0%	-10.9%, 10.5%	-68.6%, -2.0%	-65.4%, -43.0%	-89.1%, -84.2%	-62.0%, 27.7%	-72.3%, 8.9%	-17.2%, 45.8%
Mean	7.7	3.1	1.0	17.9	4.9	152.0	90.4	26.0
Observations	12,240	12,240	12,240	12,096	12,240	12,240	12,240	12,240
B. Neighborhood								
Treat*Post	−0.16	−0.04	−0.93***	−0.69**	−0.85***	−0.14**	−0.34*	−0.01
	(0.12)	(0.06)	(0.24)	(0.23)	(0.15)	(0.05)	(0.16)	(0.19)
Percent change	-14.6%	-3.8%	-60.5%	-50.1%	-57.3%	-12.7%	-28.5%	-0.9%
95% CI	-33.1%, 8.9%	-15.1%, 9.0%	-75.4%, -36.6%	-68.3%, -21.3%	-67.9%, -43.2%	-20.5%, -4.1%	-47.8%, -2.2%	-31.1%, 42.6%
Mean	4.9	2.3	0.8	9.0	2.7	141.1	69.6	31.4
Observations	12,432	12,432	12,384	12,288	12,432	12,432	12,432	12,432

Notes: Difference-in-differences Poisson regression estimates on the association of public safety and the opening of the overdose prevention center. The specifications include hexagon and month-year fixed effects. Robust standard errors clustered at the hexagon level in parentheses. The specification follows the equation in the Supplementary Material: Statistical Methods, where POST is an indicator for whether a given observation occurs after December, 2021 when the 2 safe injection sites were opened to the public. TREAT is an indicator for whether a hexagon contains one of the 2 OPCs as opposed to a comparison unit. Hence, the table shows the coefficient on the interaction between POST and TREAT, which is the estimated difference-in-differences intervention effect. The comparison group uses the 250 hexagons with the most index crimes (excluding theft) among the seven (10%) precincts with the highest index crime rates (excluding theft). Violent crimes include murder, robbery, and aggravated and simple assault. Property crimes include burglary, theft, and motor vehicle theft. Weapons refer to criminal possession of a weapon. Drugs mean the sale or possession of dangerous drugs. Crime 911 calls refer to those made to law enforcement where there was a possible crime in-progress or one has been committed. Medical calls include those needing an ambulance. Nuisance calls include 911 calls for trespass and 311 calls about homelessness (assisting a homeless person, encampment, and homeless street condition) and disorder (seeing a rodent, graffiti, dirty and unsanitary conditions, drug and drinking activity, urinating in public, and those 311 calls under the New York Police Department jurisdiction such as an abandoned vehicle and noise complaint). Panel A examines the immediate vicinity (a single hexagon around the site). Panel B inspects the neighborhood (three hexagons surrounding the site). The bottom rows exhibit the percentage change (incidence rate ratio - 1 = exp(β)-1), followed by the 95 percent confidence interval, and the pre-intervention mean count crime on the neighborhoods with OPCs and the number of observations. *p<0.05; **p<0.01; ***p<0.001.

© 2023 Chalfin A et al. *JAMA Network Open*.

eTable 7. Association Between Opening an Overdose Prevention Center and Public Safety, High Drug Arrests Comparison Sample

	Crime		Law enforcement			Calls for service		
	Violent	Property	Weapons arrests	Drug arrests	Criminal summons	Crime 911 calls	Medical 911 calls	Nuisance calls
	(1)	(2)	(3)	(4)	(5)	(6)	(7)	(8)
A. Immediate vicin								
Treat*Post	−0.08	0.01	−0.74*	−0.67**	−2.04***	−0.44	−0.71	−0.34
	(0.10)	(0.07)	(0.31)	(0.21)	(0.12)	(0.33)	(0.37)	(0.21)
Percent change	-8.0%	0.7%	-52.4%	-49.0%	-87.0%	-35.6%	-50.8%	-28.5%
95% CI	-24.2%, 11.6%	-12.5%, 16.0%	-74.2%, -11.9%	-66.1%, -23.5%	-89.8%, -83.4%	-66.1%, 22.4%	-76.4%, 2.5%	-52.7%, 7.9%
Mean	7.7	3.1	1.0	17.9	4.9	152.0	90.4	26.0
Observations	1,056	1,056	1,056	1,056	1,056	1,056	1,056	1,056
B. Neighborhood								
Treat*Post	−0.09	−0.02	−1.08***	−0.56	−0.86***	−0.21**	−0.45*	−0.44
	(0.14)	(0.08)	(0.26)	(0.29)	(0.17)	(0.08)	(0.19)	(0.24)
Percent change	-8.5%	-2.3%	-66.1%	-42.8%	-57.7%	-19.3%	-35.9%	-35.5%
95% CI	-30.0%, 19.6%	-16.3%, 14.0%	-79.8%, -43.2%	-67.4%, 0.6%	-69.5%, -41.4%	-30.4%, -6.4%	-55.5%, -7.8%	-59.8%, 3.5%
Mean	4.9	2.3	0.8	9.0	2.7	141.1	69.6	31.4
Observations	1,248	1,248	1,200	1,248	1,248	1,248	1,248	1,248

Notes: Difference-in-differences Poisson regression estimates on the association of public safety and the opening of the overdose prevention center. The specifications include hexagon and month-year fixed effects. Robust standard errors clustered at the hexagon level in parentheses. The specification follows the equation in the Supplementary Material: Statistical Methods, where POST is an indicator for whether a given observation occurs after December, 2021 when the 2 safe injection sites were opened to the public. TREAT is an indicator for whether a hexagon contains one of the 2 OPCs as opposed to a comparison unit. Hence, the table shows the coefficient on the interaction between POST and TREAT, which is the estimated difference-in-differences intervention effect. The comparison group uses the 20 hexagons with the most pre-intervention drug arrests across New York City. Violent crimes include murder, robbery, and aggravated and simple assault. Property crimes include burglary, theft, and motor vehicle theft. Weapons refer to criminal possession of a weapon. Drugs mean the sale or possession of dangerous drugs. Crime 911 calls refer to those made to law enforcement where there was a possible crime in-progress or one has been committed. Medical calls include those needing an ambulance. Nuisance calls include 911 calls for trespass and 311 calls about homelessness (assisting a homeless person, encampment, and homeless street condition) and disorder (seeing a rodent, graffiti, dirty and unsanitary conditions, drug and drinking activity, urinating in public, and those 311 calls under the New York Police Department jurisdiction such as an abandoned vehicle and noise complaint). Panel A examines the immediate vicinity (a single hexagon around the site). Panel B inspects the neighborhood (three hexagons surrounding the site). The bottom rows exhibit the percentage change (incidence rate ratio - 1 = exp(β)-1), followed by the 95 percent confidence interval, and the pre-intervention mean count crime on the neighborhoods with OPCs and the number of observations. *p<0.05; **p<0.01; ***p<0.001.

© 2023 Chalfin A et al. *JAMA Network Open*.

eTable 8. Neighborhood Sociodemographic Characteristics by Intervention Status, 5-Year Estimates

	Neighborhoods with OPCs	Neighborhoods with SSP
Race and ethnicity		
White (%)	20.5	19.1
Black (%)	16.1	36.3
Hispanic (%)	58.3	30.7
Asian (%)	3.4	9.4
Other (%)	2.4	4.2
Income levels		
Per capita (thousand dollars)	35.5	37.8
Median household (thousand dollars)	56.8	61.7
Below poverty line (%)	22.0	22.4
Other sociodemographics		
Bacherlor's degree or higher (%)	34.6	33.4
Foreign-born population (%)	35.9	31.4

Notes: The sociodemographics come from population weighted zip code level data where the OPCs (10035 and 10033) and SSPs (10002, 10009, 10010, 10018, 10027, 10302, 10451, 10459, 10301, 11207, 11208, 11221, 11212, 11217, 11412, 11435) are located Estimates based on the U.S. Census Bureau (2021). American Community Survey 5-year estimates. Retrieved from Census Reporter.

© 2023 Chalfin A et al. *JAMA Network Open*.

eFigure 6. Histogram of Preintervention Drug Arrests by Hexagonal Area, New York City

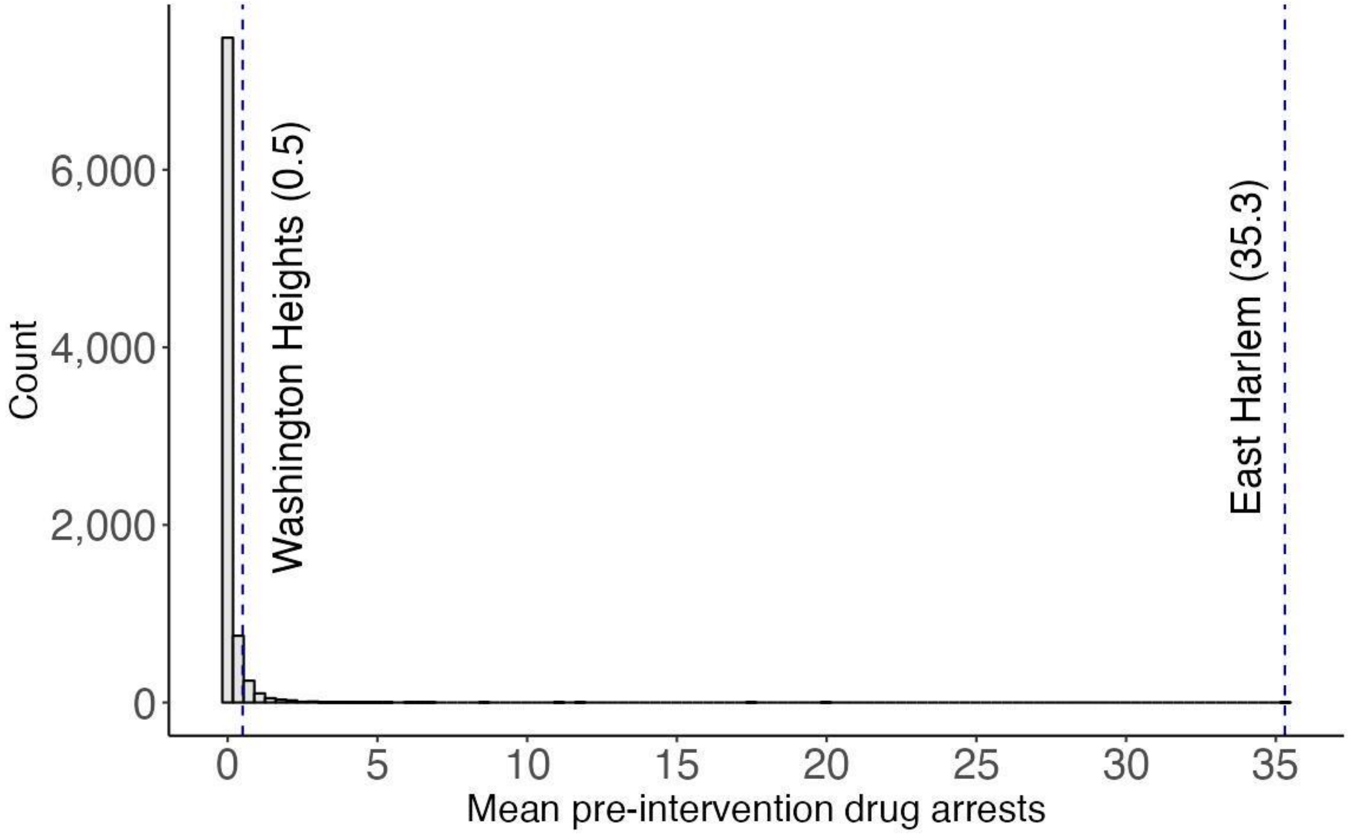

Note: Count of hexagons by their average pre-intervention drug arrests in New York City (there are 8,742 hexagons in the city). The vertical lines show the number of crimes in the OnPoint NYC East Harlem and Washington Heights overdose prevention centers.

© 2023 Chalfin A et al. *JAMA Network Open*.

eFigure 7. Public Safety Time Series by Sample, Bimonthly Hexagon Level Data

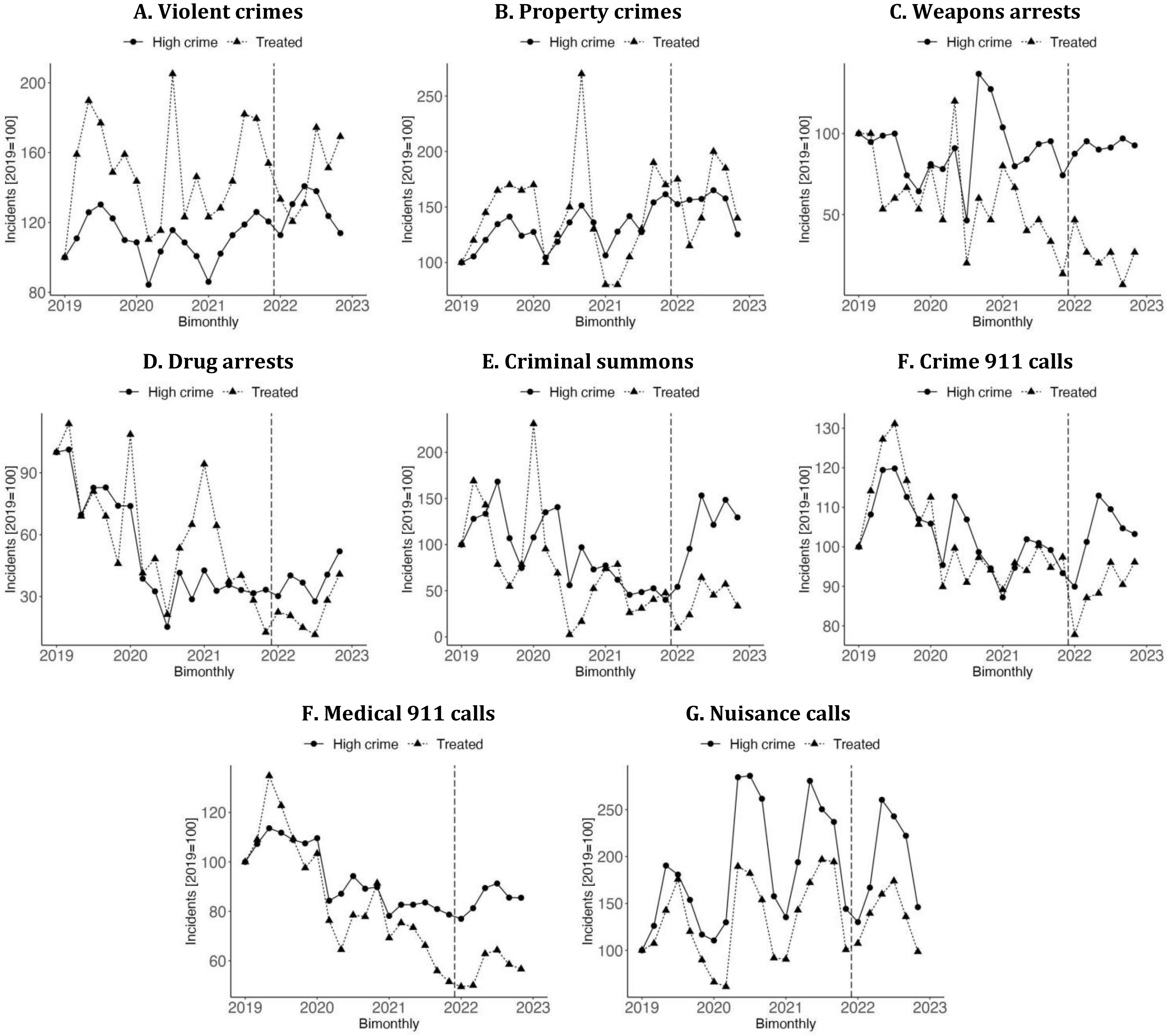

Note: The panels show proportional changes of the incidents on the average hexagon at the bimonthly level, where 2019 is the reference year. The high-crime sample was built by first identifying the top seven (10%) precincts outside of the intervention areas that had the highest number of index crimes (excluding larceny) per 100,000 residents, then selecting the 250 hexagons with the highest volume of crimes among these seven precincts. The intervention hexagon surrounds the overdose prevention center. Violent crimes include murder, robbery, and aggravated and simple assault. Property crimes include burglary, theft, and motor vehicle theft. Weapons arrests refer to criminal possession of a weapon. Drug arrests mean the sale or possession of dangerous drugs. Crime 911 calls refer to those made to law enforcement where there was a possible crime in-progress, or one has been committed. Trespass 911 calls explicitly mention this offense in the call. Medical calls include those needing an ambulance. Nuisance calls include 911 calls for trespass and 311 calls about homelessness (assisting a homeless person, encampment, and homeless street condition) and disorder (seeing a rodent, graffiti, dirty and unsanitary conditions, drug and drinking activity, urinating in public, and those 311 calls under the New York Police Department jurisdiction such as an abandoned vehicle and noise complaint).

© 2023 Chalfin A et al. *JAMA Network Open*.

eFigure 8. Spatial Distribution of the High Crime Hexagons Sample

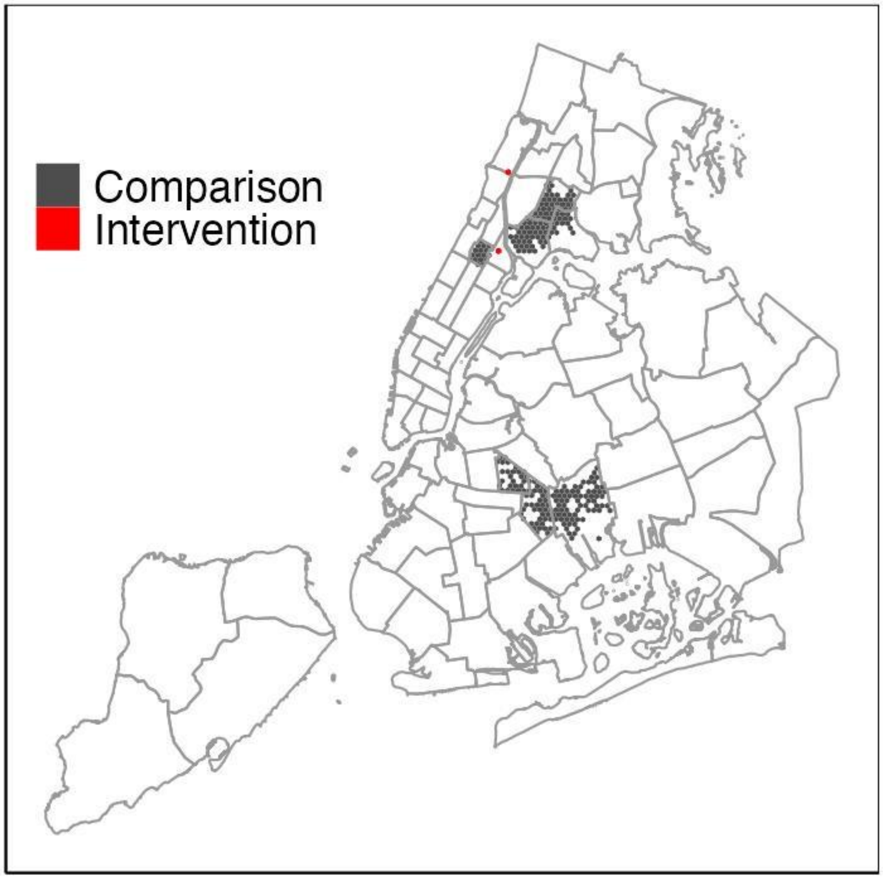

Note: To reduce concerns of selecting areas with high pedestrian traffic inflating the volume of crime (e.gr., Midtown Manhattan receives millions of tourists, so its crime risk is low relative to the actual foot traffic rather than residents exposed to such crimes), an alternative process was done to select high crime hexagons sample. Specifically, the sampling process first selected the top seven (10%) precincts outside the intervention areas with the highest index crimes (excluding larceny) per 100 thousand residents. Larceny was excluded as it is highly correlated to foot traffic. Then, it was chosen the 250 hexagons with the highest volume of crimes among these seven precincts.

© 2023 Chalfin A et al. *JAMA Network Open*.

eFigure 9. Spatial Distribution of the High Drug Arrests Hexagons Sample

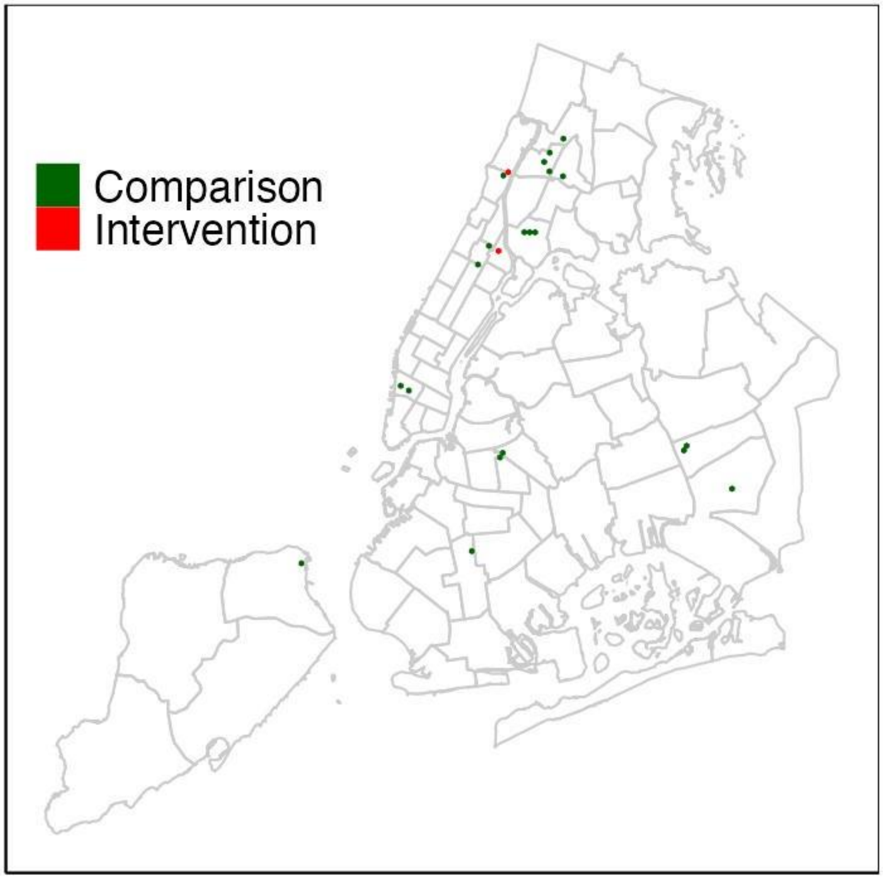

Note: This sample selected the 20 hexagons with the most pre-intervention mean drug arrests excluding areas in the police precincts where the overdose prevention centers are located. This process provides a sample with a similar volume of drug arrests to the intervention areas.

© 2023 Chalfin A et al. *JAMA Network Open*.

eFigure 10. Poisson Event Study Design Estimates on Public Safety, High Crime Sample

I. Immediate vicinity

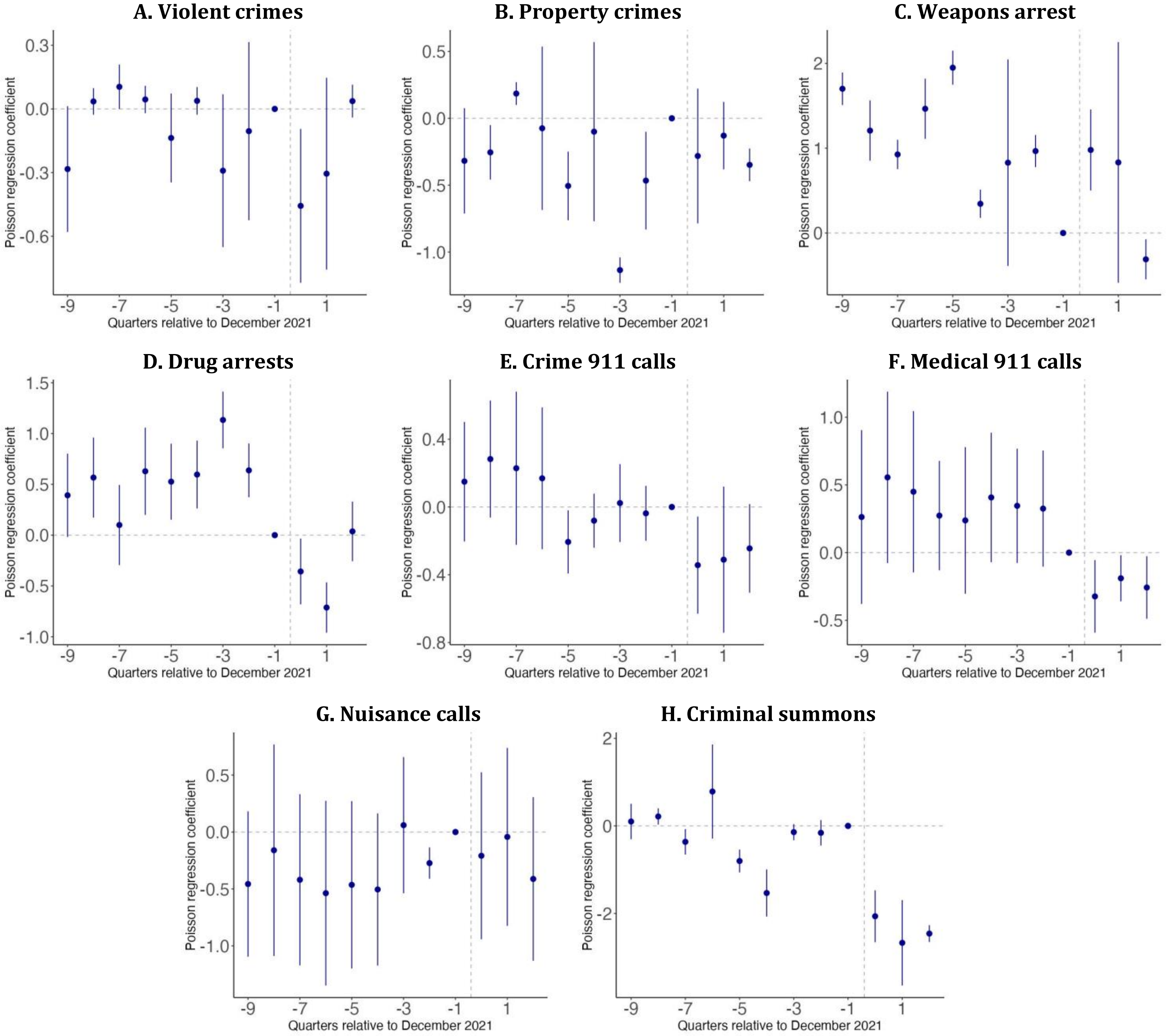

© 2023 Chalfin A et al. *JAMA Network Open*.

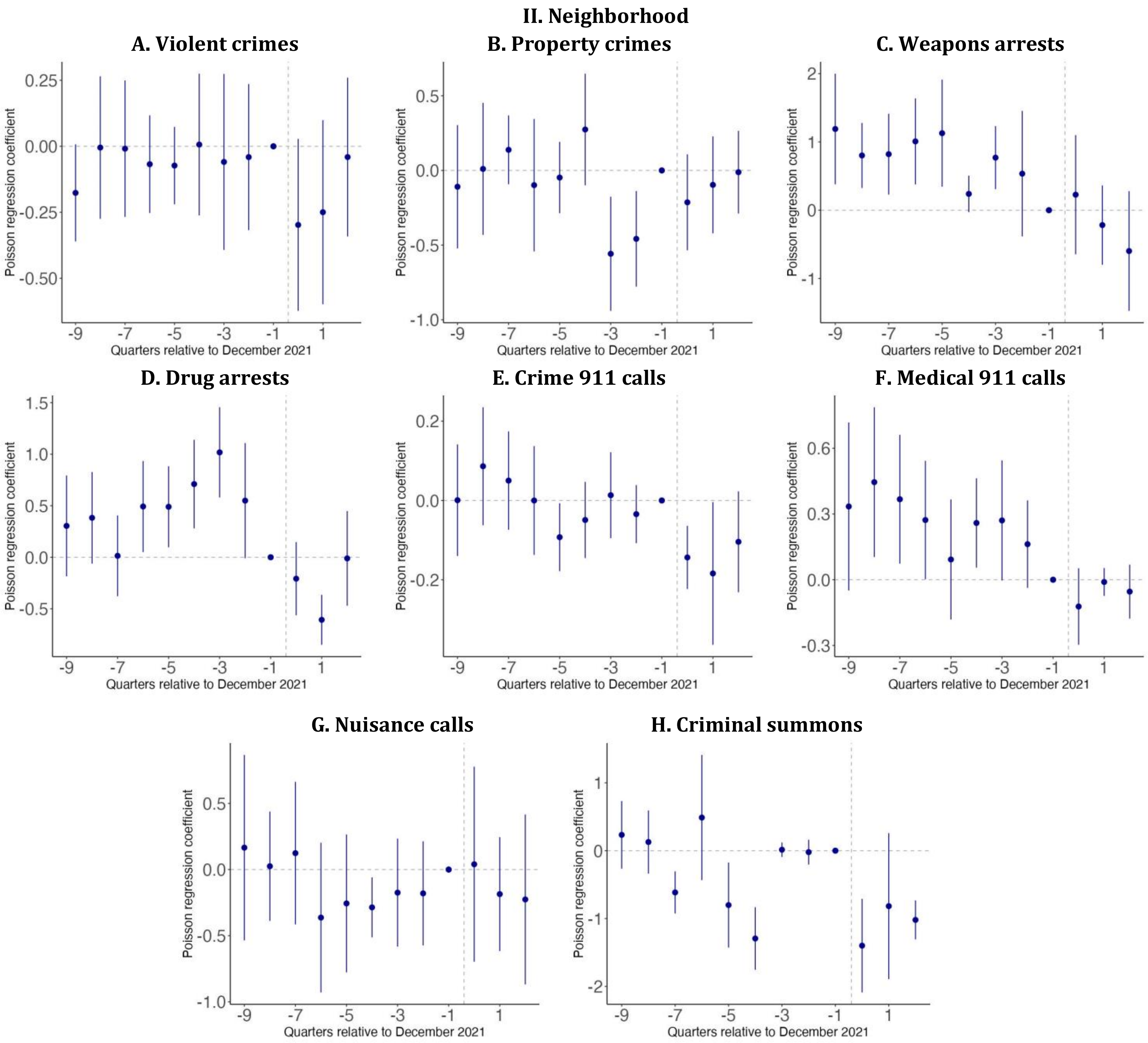

Note: The panels show the event study design estimates using a Poisson regression. Each estimate represents a four-month bin relative to the opening of the overdose prevention centers (December 2021). The models include hexagon and month-year fixed effects. The standard errors are clustered at the hexagon level using the following specification: $y_{it} = \omega_i + \sigma_t + \sum_{\tau=-q}^{m} \beta_\tau D_{it} + e_{it}$. The comparison group uses the 250 hexagons with the most index crimes (excluding theft) among the seven (10%) precincts with the highest index crime rates (excluding theft). Violent crimes include murder, robbery, and aggravated and simple assault. Property crimes include burglary, theft, and motor vehicle theft. Weapons arrests refer to criminal possession of a weapon. Drug arrests mean the sale or possession of dangerous drugs. Crime 911 calls refer to those made to law enforcement where there was a possible crime in-progress, or one has been committed. Trespass 911 calls explicitly mention this offense in the call. Medical calls include those needing an ambulance. Nuisance calls include 911 calls for trespass and 311 calls about homelessness (assisting a homeless person, encampment, and homeless street condition) and disorder (seeing a rodent, graffiti, dirty and unsanitary conditions, drug and drinking activity, urinating in public, and those 311 calls under the New York Police Department jurisdiction such as an abandoned vehicle and noise complaint).

© 2023 Chalfin A et al. *JAMA Network Open*.

eFigure 11. Poisson Event Study Design Estimates on Public Safety, High Drug Arrests Sample

I. Immediate vicinity

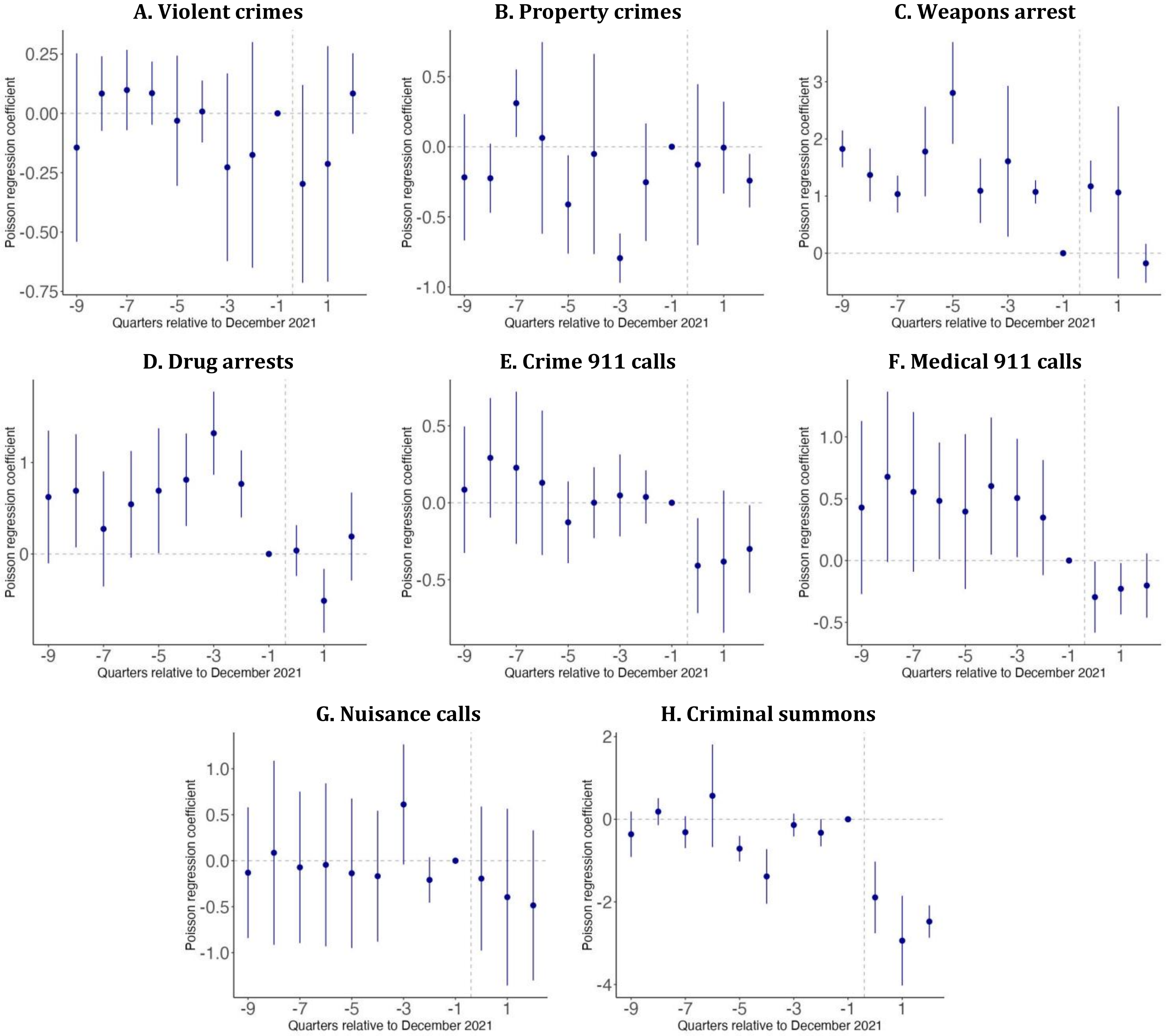

© 2023 Chalfin A et al. *JAMA Network Open*.

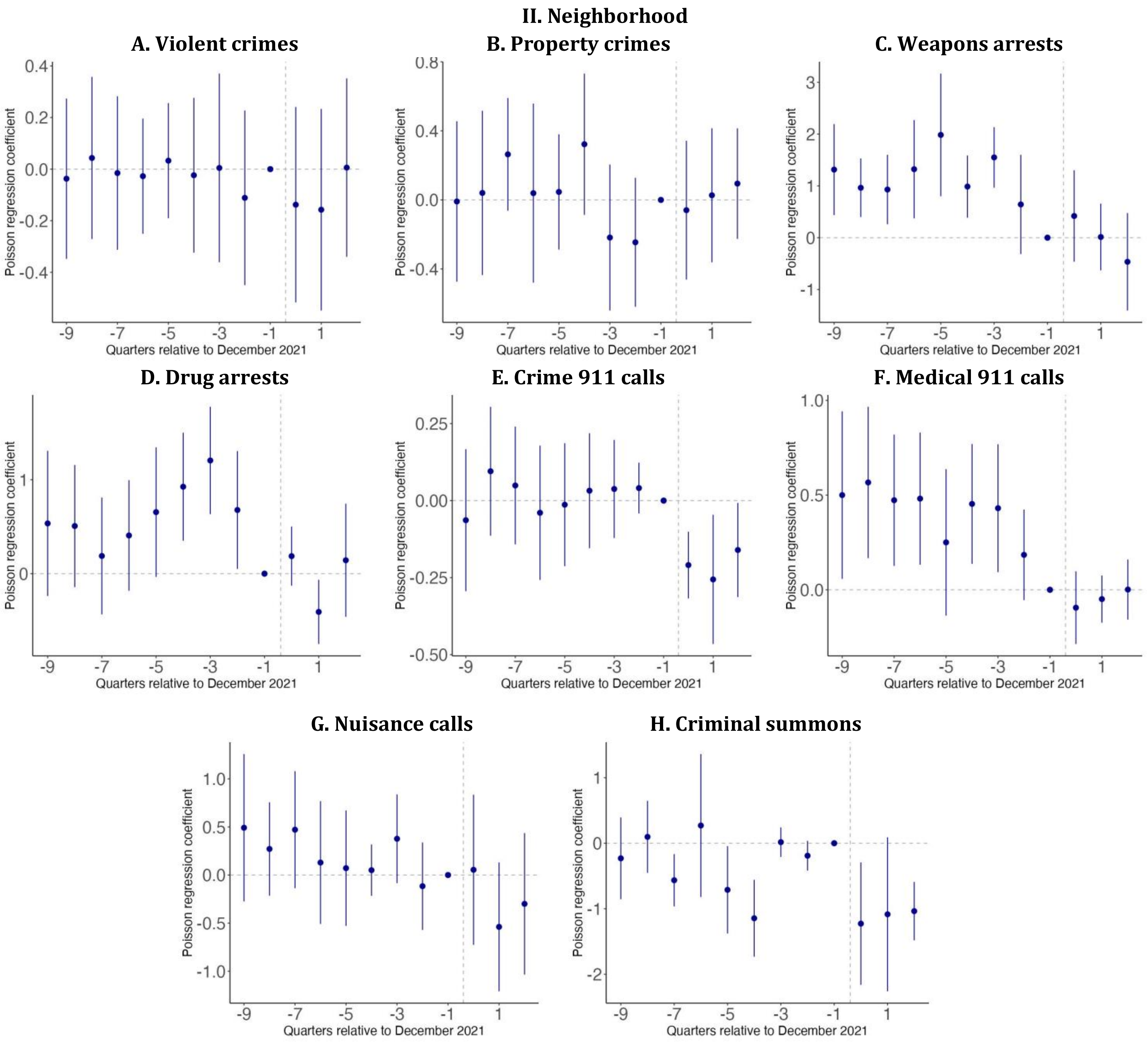

Note: The panels show the event study design estimates using a Poisson regression. Each estimate represents a four-month bin relative to the opening of the overdose prevention centers (December 2021). The models include hexagon and month-year fixed effects. The standard errors are clustered at the hexagon level using the following specification: $y_{it} = \omega_i + \sigma_t + \sum_{\tau=-q}^{m} \beta_\tau D_{it} + e_{it}$. The comparison group uses the 20 hexagons with the most pre-intervention mean drug arrests excluding areas in the police precincts where the overdose prevention centers are located. Violent crimes include murder, robbery, and aggravated and simple assault. Property crimes include burglary, theft, and motor vehicle theft. Weapons arrests refer to criminal possession of a weapon. Drug arrests mean the sale or possession of dangerous drugs. Crime 911 calls refer to those made to law enforcement where there was a possible crime in-progress, or one has been committed. Trespass 911 calls explicitly mention this offense in the call. Medical calls include those needing an ambulance. Nuisance calls include 911 calls for trespass and 311 calls about homelessness (assisting a homeless person, encampment, and homeless street condition) and disorder (seeing a rodent, graffiti, dirty and unsanitary conditions, drug and drinking activity, urinating in public, and those 311 calls under the New York Police Department jurisdiction such as an abandoned vehicle and noise complaint).

© 2023 Chalfin A et al. *JAMA Network Open*.

eFigure 12. Difference-in-Differences Estimates on Nuisance Calls by Model Specification

A. Immediate vicinity

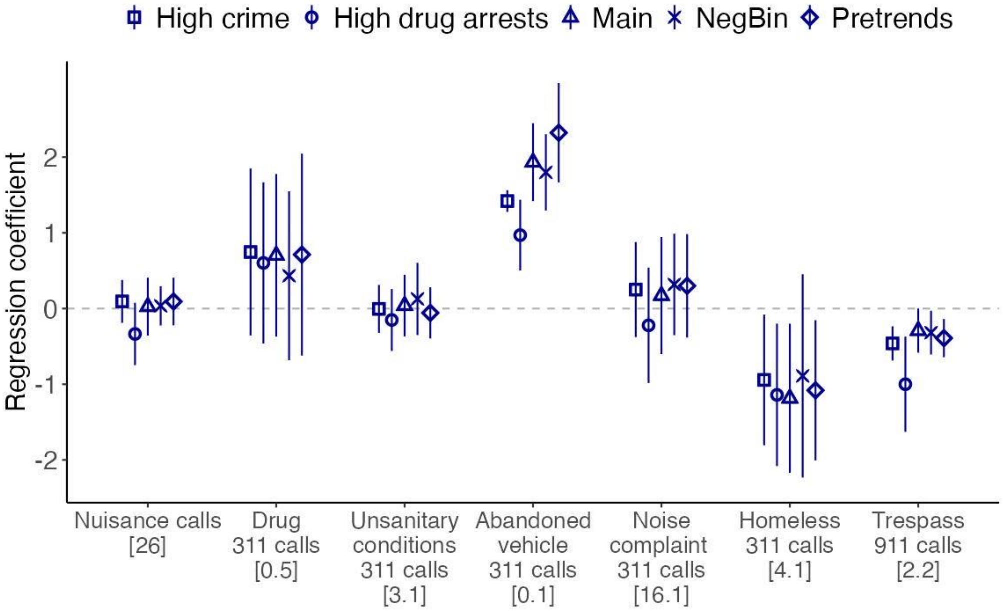

B. Neighborhood

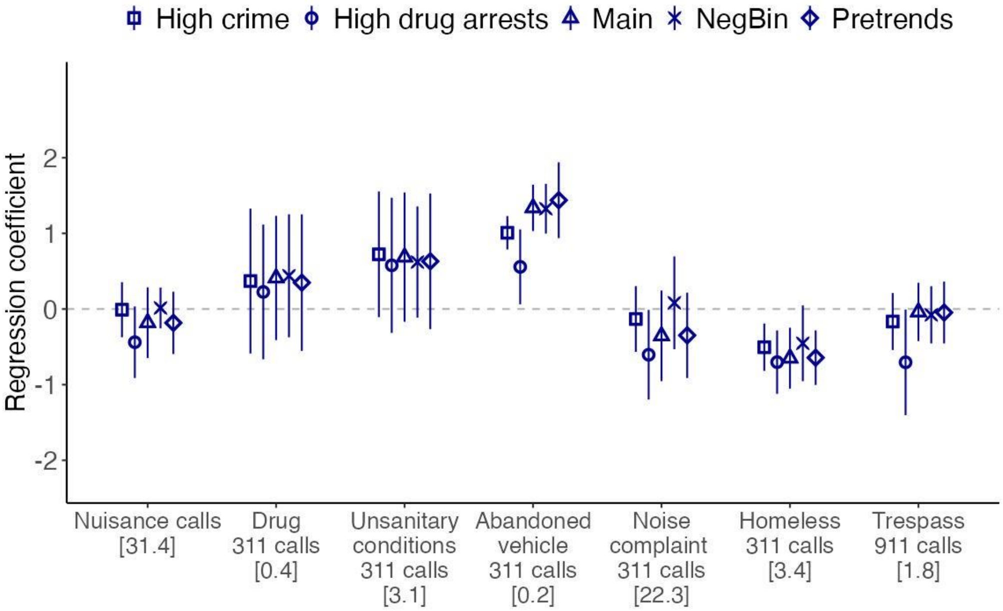

Note: Difference-in-differences estimates on the association of public safety and the opening of the overdose prevention centers by model specification. All specifications include hexagon and month-year fixed effects and robust standard errors clustered at the hexagon level. Panel A examines the immediate vicinity (a single hexagon around the site). Panel B inspects the neighborhood (three hexagons surrounding the site). The high crime sample estimates a Poisson regression using as the comparison group the 250 hexagons with the most index crimes (excluding theft) among the seven (10%) precincts with the highest index crime rates (excluding theft). The high drug arrests sample estimates a Poisson regression using the comparison group the 20 hexagons with the most pre-intervention drug arrests across New York City. The main (preferred) specification computes a Poisson regression using the 17 syringe service programs as the comparison group. The NegBin specification computes a Negative Binomial regression using the 17 syringe service programs as the comparison group. The pre-trends specification computes a Poisson regression, including hexagon and month-year fixed effects and controls for two four-month bin pre-intervention indicator variables. The bottom rows exhibit the pre-intervention mean on the intervention in squared brackets. Nuisance calls include drug, unsanitary conditions, abandoned vehicles, noise complaints, and homeless 311 calls and trespass 911 calls. Drug-related 311 calls refer to drug and drinking activity and loose syringes. Unsanitary conditions comprise calls about seeing a rodent, graffiti, dirty and unsanitary conditions, and urinating in public. Abandoned vehicle and noise complaints are calls handled by the New York Police Department. Homeless calls include those related to assisting a homeless person, encampment, and homeless street conditions. Trespass 911 calls explicitly mention this offense in the call.

© 2023 Chalfin A et al. *JAMA Network Open*.
