# Supplementary material for: Overdose Prevention Centers, Crime, and Disorder in New York City
Source: JAMA Netw Open. 2023 Nov 13;6(11):e2342228. doi: 10.1001/jamanetworkopen.2023.42228 (PMC10644216; doi:10.1001/jamanetworkopen.2023.42228)
Supplement: Supplement 2. — Data Sharing Statement [file jamanetwopen-e2342228-s002.pdf]

Data Sharing Statement

Chalfin. Overdose Prevention Centers, Crime, and Disorder in New York City. *JAMA Netw Open*. Published November 30, 2023. doi:10.1001/jamanetworkopen.2023.42228

Data
Data available: Yes
Data types: Deidentified participant data, Data (not involving human participants), Data dictionary, Other (please specify)
Additional Information: Municipal administrative data.
How to access data: https://data.cityofnewyork.us/browse?Dataset-Information_Agency=Police+Department+%28NYPD%29
When available: With publication

Supporting Documents
Document types: Statistical/analytic code
How to access documents: Please direct requests to david.mitre@uconn.edu, who will make the analytic code publicly available.
When available: With publication

Additional Information
Who can access the data: The data is in the public domain and the analytic code will be made available to any person.
Types of analyses: The data is already in the public domain, available for any purpose.
Mechanisms of data availability: The data is already available to any person, and will not be provided with investigator support.
Any additional restrictions: None.
